# Community level youth-led interventions to improve maternal-neonatal outcomes in low- and middle-income countries: A systematic review of randomised trials

**DOI:** 10.7189/jogh.13.04168

**Published:** 2023-12-27

**Authors:** Tonya MacDonald, Nadia Rehman, Maya Stevens-Uninsky, Naharin Sultana Anni, Rachel Liu, Elizabeth K Darling, Saara Greene, Sandra Moll, Lawrence Mbuagbaw

**Affiliations:** 1 Department of Health Research Methods, Evidence and Impact, McMaster University, Hamilton, Ontario, Canada; 2 Department of Global Health, McMaster University, Hamilton, Ontario, Canada; 3 McMaster Midwifery Research Centre, McMaster University, Hamilton, Ontario, Canada; 4 Department of Obstetrics and Gynecology, McMaster University, Hamilton, Ontario, Canada; 5 Faculty of Social Sciences, School of Social Work, McMaster University, Hamilton, Ontario, Canada; 6 School of Rehabilitation Science, McMaster University, Hamilton, Ontario, Canada; 7 Department of Anesthesia, McMaster University, Hamilton, Ontario, Canada; 8 Department of Pediatrics, McMaster University, Hamilton, Ontario, Canada; 9 Biostatistics Unit, Father Sean O’Sullivan Research Centre, St. Joseph’s Healthcare, Hamilton, Ontario, Canada; 10 Centre for the Development of Best Practices in Health, Yaoundé Central Hospital, Yaoundé, Cameroon; 11 Division of Epidemiology and Biostatistics, Department of Global Health, Stellenbosch University, Cape Town, South Africa

## Abstract

**Background:**

Evidence on the effectiveness of youth-led interventions for improving maternal-neonatal health and well-being of women and gender diverse childbearing people in low-income and middle-income countries (LMICs) is incomplete. We aimed to summarise the evidence on whether community level youth-led interventions can improve maternal and neonatal outcomes in LMICs.

**Methods:**

We included experimental studies of youth-led interventions versus no intervention, standard care, or another intervention. Participants were women and gender diverse childbearing people during antepartum, intrapartum, and postpartum periods. MEDLINE, Embase, CINAHL, Global Health, Web of Science, and Cochrane Library, and grey literature were searched to January 2023. All interventions addressing and targeting maternal-neonatal health and well-being that were youth-led and community level were included. Primary outcomes of interest were maternal death and neonatal death. We excluded based on population, intervention, comparison, and outcome (PICO) and design. Two reviewers independently extracted key information from each included study and assessed risk of bias. Random-effects meta-analysis was performed where there were sufficient data. The certainty of evidence was assessed using Grading of Recommendations, Assessment, Development and Evaluation (GRADE). A narrative synthesis was done for results that could not be pooled.

**Results:**

Of the 8054 records retrieved, four trials (21 813 enrolled participants) met the inclusion criteria. The Cooperative for Assistance and Relieve Everywhere, Inc. (CARE) Community Score Card intervention compared to standard reproductive health services control did not significantly improve Antenatal Care coverage (difference-in-differences estimate β = 0.04; 95% confidence interval (CI) = -0.11, 0.18, *P* = 0.610; one study, low certainty of evidence). The multi-component social mobilisation interventions compared to standard of care had no effect on adolescent/youth pregnancy (adjusted odds ratio estimate = 1.08; 95% CI = 0.87, 1.33; three studies; low certainty of evidence).

**Conclusions:**

Youth-led interventions in LMICs did not show a significant improvement in maternal outcomes. More studies are required to make more precise conclusions.

**Registration:**

PROSPERO: CRD42021288798.

Everyday globally hundreds of women and gender diverse childbearing people, and thousands of neonates (newborns) die from preventable causes related to pregnancy or childbirth. In our communities, and within countries and across nations, global maternal and neonatal survival are inequitable.

Maternal and neonatal death are embedded within a context of inequity, and fueled by a lack of human, financial, and infrastructural resources especially in countries with the highest maternal mortality rates (MMRs) and neonatal mortality rates (NMRs). In low-income and middle-income countries (LMICs) and least developed countries (LDCs) (i.e., low-resource settings confronting severe structural barriers to sustainable development), there are extremes of inequity in survival [1]. Currently, the MMR point estimate for Europe and Northern America is 12 maternal deaths per 100 000 live births; for sub-regions of Asia and Western Africa there is an MMR point estimate range of 69 to 151 [2]. The MMR point estimate is highest for LDCs at 415 maternal deaths per 100 000 live births; and remains overall highest in the region of Sub-Saharan Africa with an MMR point estimate as high as 542 maternal deaths for 100 000 live births [2]. Globally, in 2019 the average rate of neonatal deaths was 17 deaths per 1000 live births [3]. Like maternal death, there are also widespread regional disparities in neonatal and under-five chances of survival, with the region of Sub-Saharan Africa having the highest under-five mortality rate in the world [3].

Renewed by the United Nation’s Sustainable Development Goals (SDGs), nations are committed to working to end preventable maternal mortality and neonatal death globally. With the goal of eliminating extremes of inequity in global maternal and neonatal survival by 2030, we aim for an average global target MMR of less than 70 maternal deaths per 100 000 live births, and a supplementary national target that will have no country with an MMR greater than 140 deaths per 100 000 live births [4]. We are also aiming for an average global target of fewer than 25 neonatal deaths per 1000 live births [3].

Many LMICs that are working to reduce their rates of maternal and neonatal deaths, also have proportionately high youth populations. Youth- defined by the United Nations (UN) – as people aged 15-24 years [5] have strength in their numbers. Could youth, an often marginalised, disregarded and voiceless group [6,7] be an untapped, available, and willing resource to improve maternal health and well-being in their communities? Globally there are many examples of youth-focussed and youth-led interventions in health, governance and democracy, economic development, and education [8]. In youth-led interventions, proponents not only regard youth as important agents of social awareness and transformation but cite “higher levels of creativity and energy among youth and a higher potential to introduce innovations compared to adults” [9].

Youth-led interventions, such as those in health, education, governance and democracy, and economic development aim to benefit the community whilst engaging the community’s future leaders [9-11]. Meaningful youth involvement has the potential to broaden social and structural contexts that strengthen health and well-being [9-11]. With specific regard to maternal-neonatal health, youth-led interventions hold the potential to promote positive health behaviours, strengthen community understanding of barriers to health services access, support educational activities related to reproductive and sexual health, and advocate for health equity among marginalised populations such as women, girls, youth, two-spirit, and lesbian, gay, bisexual, transgender, queer and/or questioning, intersex, asexual+ (2SLGBTQIA+) folks, for example.

This review focusses on youth-led interventions for improving maternal-neonatal health and well-being in communities of LMICs. The appraisal of the evidence was completed to identify knowledge gaps and potential solutions regarding youth-led interventions in LMICs. Ultimately the evidence may help future community planning and policy developments that aim to improve maternal-neonatal health and well-being and to include youth engagement in maternal-neonatal health initiatives in LMICs. At the time of this systematic review, there was no systematic review that assessed the effects of community level youth-led interventions on maternal and neonatal health and well-being.

The objectives of this systematic review were to appraise and to summarise the evidence on the effects of community level youth-led interventions for improving maternal-neonatal health and well-being compared with no interventions or another intervention in LMICs.

## METHODS

The methods are detailed in the published protocol [12] and reported in brief here.

### Eligibility criteria

#### Types of studies

Study designs eligible for inclusion were randomised control trials (RCTs), quasi-randomised trials (QRTs), and cluster randomised trials (CRTs). Included studies needed to evaluate maternal-neonatal health and well-being associated with or because of the implementation of community level youth-led interventions and aimed at members of the community. Only studies in LMICs were included.

#### Population/participants

We included studies of maternal health outcomes of women and gender diverse childbearing people at different maternal periods who received a youth-led intervention, and where there was evaluation of maternal or neonatal health and well-being.

#### Interventions

Eligible interventions needed to be youth-led, where youth were people 15-24 years old as per the UN definition [5]. Included studies had youth involved in leading or delivering all or part of the interventions. It was not necessary that interventions be youth-designed or initiated. Interventions could have included clinical, educational, behavioural, or policy interventions, single, bundled, or multi-component, or other types of interventions that may have been non-specific, multi-pronged, preventative, or therapeutic in nature.

#### Comparators

The comparator in eligible RCTs was defined as no intervention, usual or standard care, or another intervention.

#### Outcomes

The primary outcomes of interest were maternal death and neonatal death. Secondary outcomes were antenatal care coverage, births attended by skilled health personnel, adolescent birth rate, stillbirth, postpartum haemorrhage, and maternal near-miss. Definitions of the outcomes are in Appendix S1 in the **Online Supplementary Document**. We included studies with any duration of follow-up and with no restrictions on timing.

### Information sources

We conducted a comprehensive and exhaustive literature search within each of the following electronic databases from inception to 30 January 2023: MEDLINE (1946 to present), Excerpta Medica Database (Embase) (1974 to present), Cumulative Index to Nursing and Allied Health Literature (CINAHL) (1981 to present), Latin American and Caribbean Health Sciences literature (LILACS) (1982 to present), Global Health (1910 to present), Web of Science (1976 to present), and Cochrane Central Register of Controlled Trials (CENTRAL) (1996 to present). We also searched the following trial registers up to 28 January 2023: ClinicalTrials.gov, World Health Organization (WHO) International Clinical Trials Registry Platform (ICTRP), and International Standard Randomized Controlled Trial Number (ISRCTN).

We searched the websites of databases with a regional focus or of key health organisations in the area of health (the UN website WHO Health Evidence Network (WHO HEN) and the Centers for Disease Control and Prevention (CDC) website) and conducted electronic searches of the following grey literature databases using search strategies adapted from our MEDLINE search strategy up to 26 January 2023: Directory of Open Access Journals (DOAJ) and the National Institute of Health Research (NIHR).

### Search strategy

The search strategy was developed through guidance of Health Sciences Librarians with expertise in systematic review searching. Our strategy was peer reviewed by the authors of the systematic review and by an information specialist by making use of the Peer Review of Electronic Search Strategies (PRESS) [13]. The databases were searched without date or language restrictions. Authors of relevant reports were contacted by email to locate other relevant published or unpublished studies. We hand searched reference lists of all eligible study reports to check for additional studies that may have been missed. Our search was completed between January 2022 and March 2022, and was updated in January 2023. The search strategy for each database is included in Appendix S2 in the **Online Supplementary Document**. 

### Selection process

Citations identified from the literature searches and reference lists were first run through the SR-accelerator (SRA Bond University, Robina, Australia) [14] for deduplication, then imported to EndNote (EndNote, Philadelphia PA, USA) [15] where any further duplicates were removed. Titles, abstracts, and full texts were reviewed using a piloted, standardised screening form in DistillerSR software (DistillerSR Inc, Ottawa Ontario, Canada) [16]. Relevant articles were discussed by two reviewers (in pairs, as TMA-NSA, NR-TM, MSU-RL) to make a final decision about study inclusion or exclusion. We recorded the reason for exclusion at the full-text screening. When multiple reports arising from the same study were identified, we matched these based on study name, author names, and study locations, dates, and sample characteristics. Data were extracted only from the report that had the most complete information for the review (e.g., based on reported outcomes, most relevant analysis). We contacted corresponding (or second) authors by email with up to two attempts to obtain or confirm relevant information from study investigators. Disagreements were resolved by consensus or by involving an adjudicator.

### Data collection process

Two reviewers extracted data independently and in duplicate to collect information from each included study using piloted forms. We resolved discrepancies through discussion and involved another review author to reach consensus. We contacted study investigators to resolve uncertainties or if data were missing. The two reviewers entered data into Review Manager (RevMan 5) software (RevMan Cochrane Collaboration, London, England) [17] and checked for accuracy. We calculated agreement on screening using the kappa (κ) statistic [18].

### Data items

Data were extracted on bibliometric information, study characteristics, and the primary and secondary outcomes (Appendix S3 in the **Online Supplementary Document**). If data were available at multiple time points, we only extracted data from the final study follow-up. We intended to include data from outcomes that matched either our primary or secondary outcomes of interest in meta-analyses. Intention-to-treat data were extracted where available. When trial authors reported pregnancy of adolescents or youth occurring during the trial or at the final follow-up period, we assumed that the majority would go on to give birth and used it as a proxy for adolescent (or youth) birth.

### Risk of bias

We used the Cochrane risk-of-bias tool for randomised trials (RoB 2) [19] and RoB 2 for CRTs [20]. The tools included five domains and signaling questions as dimensions of bias assessment [20] and six additional items for CRTs (selection, performance, detection, attrition, reporting, and analysis biases) as additional sources of bias [21,22]. Outcomes were judged to be at “low risk of bias”, having “some concerns”, or “high risk of bias” [19]. RoB tables were filled independently by the two reviewers for each of the included studies.

### Effect measures

For studies where data were appropriate for synthesis of dichotomous outcomes, we determined treatment effect by using adjusted odds ratios (aOR) or risk ratios (RR) with 95% confidence interval (CI). For studies where data for continuous outcomes could be synthesised, we planned to use weighted mean differences or standardised mean differences (95% CI). For studies where data were non-quantitative, we presented these data descriptively or narratively.

### Synthesis methods

Random-effects meta-analysis was performed where studies were sufficiently similar. Results that could not be pooled were synthesised narratively.

We assessed all included studies for clinical heterogeneity i.e., sufficient similarity across studies in terms of participants, interventions, comparisons, and outcomes. Given the complexity of the interventions being investigated, we attempted to categorise the included interventions along four dimensions: setting(s) of intervention, components of intervention, youth implication in various components of intervention, and outcomes assessed.

We used cluster-adjusted estimates from CRTs where available. If there were large amounts of missing data, to determine the impact of missing data we planned to conduct a sensitivity analysis. If there were issues about unit of analysis, where appropriate we adjusted for the sample sizes of included CRTs by following Cochrane-described methods of using an estimate of the interclass correlation co-efficient (ICC) [22].

We synthesised evidence narratively as well as graphically using a forest plot. We created one forest plot for the outcome of adolescent/youth pregnancy. We also developed a Summary of findings (SoF) table using GRADEproGDT (GRADEproGDT, McMaster University and Evidence Prime, Hamilton ON, Canada) [23]. This table comprised summaries of the estimated intervention effect and the number of participants and studies for outcomes and included justifications for our Grading of Recommendations, Assessment, Development and Evaluation (GRADE) assessments.

For included studies that were sufficiently similar, we pooled them in a meta-analysis and evaluated statistical heterogeneity using a random effects model and the generic inverse variance method. We conducted a visual inspection of the results to identify statistical heterogeneity. We also used the χ^2^ test with a significance level of alpha = 0.10, and the *I*^2^ test to evaluate the extent of inconsistency of the pooled studies’ results. Statistical analysis was done using the RevMan 5 software [18].

We planned to consider possible sources of heterogeneity and potential subgroups for analysis that included age, duration of intervention, male versus female youth-led interventions, urban versus rural setting.

### Reporting bias assessment

We planned to assess publication bias based on funnel plot asymmetry and Egger’s test. To assess outcome reporting bias, we compared the outcomes specified in trial protocols with the outcomes reported in the corresponding trial publications; or we assessed the outcomes reported in the methods sections with those in the results sections of the trial publications.

### Certainty assessment

We judged the overall certainty of the evidence for each outcome using the GRADE approach [24]. Two reviewers assessed the certainty of the evidence across the domains of risk of bias, precision, consistency, publication bias (internal validity), and directness (external validity). For each outcome, we rated the certainty of evidence as either high, moderate, low, or very low. Certainty of evidence rated as: high indicates that further research is very unlikely to change the confidence in the effect estimate for the outcome; moderate that further research is likely to have an important impact on the confidence in the effect estimate and may change the estimate; low that future research is very likely to have an important impact on the confidence of the effect estimate and is likely to change the estimate; and very low indicates that the estimate of the effect is very uncertain [24]. We used GRADEproGDT software [23] to prepare the SoF tables.

### Participant and public involvement statement, ethics approval

There was no participant or public involvement in the design, conduct or reporting of our systematic review or in its dissemination. This meta-analysis and narrative synthesis did not involve individual participant data. Institutional review board approval was not required.

## RESULTS

### Study selection

**Figure 1:**
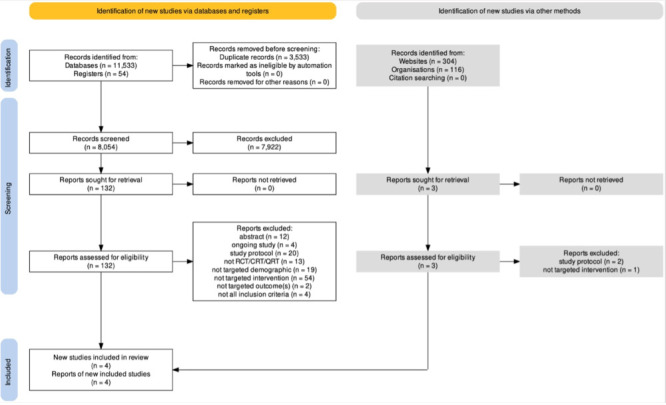
PRISMA flow diagram. RCT – randomised controlled trial.

We found 11 587 records through our searches and screened 8054 records, after deduplication. We excluded 7922 records by title/abstract screening. Full text screening was done on 132 records of which we excluded 128 records. In January 2023, the search was updated but yielded no new studies. Taken together, two reviewers extracted data from four included studies. Agreement on screening was almost perfect (κ = 0.83) [18] The results of the search and study selection process are illustrated in our Preferred Reporting Items for Systematic Reviews and Meta-Analyses (PRISMA) flow diagram in **Figure 1** [25].

### Study characteristics

We included four single-center cluster randomised control studies published between 2007 and 2017 and conducted in rural areas of Malawi [26], Zimbabwe [27], Tanzania [28], and South Africa [29]. The characteristics of the included studies are shown in **Table 1** and Appendix S4 in the **Online Supplementary Document**. The included studies only referred to “women” and none referred to gender diverse or non-binary genders within their study populations. A summary of the main intervention components is described using the Template for Intervention Description and Replication (TIDieR) checklist [31] and shown in Table S1 in the **Online Supplementary Document**. This summary may assist readers to compare characteristics of the interventions and consider the feasibility for implementation in their own setting, or to highlight missing details about the interventions, for example [32,33].

**Table 1:** Characteristics of included studies

Study ID				
Author (year)	Gullo (2017) [26]	Cowan (2010) [27]	Ross (2010) [28]	Jewkes (2008) [29]
Study design	CRT	CRT	CRT	CRT
**Population**				
Country	Malawi	Zimbabwe	Tanzania	South Africa
Setting	Health facilities and community programs	In/out-school programs and clinics	Schools, health clinics and community	Community
Sex of all enrolled/all trial outcomes, n (%)	Female, 1300 (100)	Female, 2593 (56)	Female, 4116 (44.7)	Female, 1416 (50)
Females*, n (%)	346 (53)	204 (15.1)	545 (37.2)	137 (26)
Age* range in years (%) or mean (SD)	15-19 (18.0), 20-24 (30.7), missing data	14.9 (SD not reported)†	14-17 (98.3), >18 (1.7)	15-17 (43.6), 18-19 (36.1), 20-21 (16.3), 22-26 (4.0)
Follow-up in months	24	36 and 48	36	24
**Intervention**				
Intervention name	CARE Community Score Card	Regai Dzive Shiri	MEMA kwa Vijana	The South African Stepping Stones HIV prevention program
Identification	Facility records	hCG pregnancy test	hCG pregnancy test	Self-reported
Dose and delivery by	Every six to seven months over 24 mo, facility-based providers, CHWs, HSAs, community members including youth	24 sessions over four weeks, youth PPEs, parents, community stakeholders, clinic staff	40-min sessions/mo over 12 mo, youth age 14 to >18, teachers, CHWs	50 h over six-to-eight-week period, youth facilitators age 16 to >23
**Outcomes**				
Trial outcomes	Contraceptive use, pregnancy, postnatal care use service satisfaction	Primary: HIV and HSV-2 infections, Secondary: sex knowledge, attitudes, behaviours, and pregnancy prevalence	Primary: HIV incidence, HSV2 prevalence, secondary: biological, behavioural, attitudinal, knowledge outcomes, and pregnancy	Primary: HIV incidence, Secondary: HSV2 incidence, reported sexual practices, depression, substance misuse, and unwanted pregnancy
Review outcomes	ANC coverage	Adolescent/youth pregnancy	Adolescent/youth pregnancy	Adolescent/youth pregnancy
Effect size	DiD = 0.04 (95% CI = -0.11, 0.18)	aOR = 0.92 (95% CI = 0.7, 1.19)	aOR = 1.09 (95% CI = 0.85, 1.4)	aOR = 1.45 (95% CI = 0.92, 2.28)

aOR – adjusted odds ratio, ANC – antenatal care, CARE – Cooperative for Assistance and Relieve Everywhere, Inc, CHW – community health worker, CI – confidence interval, CRT – cluster randomized trial, DiD – difference-in-differences, HIV – human immunodeficiency virus, HSV2 – herpes simplex virus-2, hCG – human chorionic gonadotropin, mo – month, NR – not recorded, PPE – professional peer educator, SD – standard deviation

*Only for review outcomes of ANC coverage or adolescent/youth pregnancy.

†Data from Cowan et al. (2008) [30].

### Risk of bias in studies

**Figure 2:**
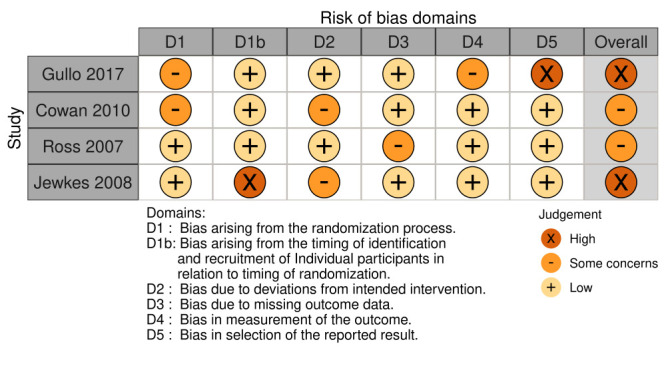
Risk of bias summary for included studies. SE – standard error, CI – confidence interval.

We assessed risk of bias for the outcome of ANC coverage in one included study [26], and the surrogate outcome of adolescent/youth pregnancy measured in three of the four studies [27-29]. In two of these studies [26,29], there were concerns of overall high risk of bias. Concerns related to bias arising during identification-recruitment and in relation to timing of randomisation, and bias in the selection of reported results. We had some concerns about the randomisation process, and with deviations from the intended intervention. A summary of these assessments is provided in **Figure 2**. To prepare our table, we used robvis [34] (robvis, Bristol, England), a web app tool for visualising risk-of-bias assessments.

### Results of individual studies

None of the studies evaluated our primary outcome measures of maternal death or neonatal death. Likewise, none of the studies assessed secondary outcome measures of births attended by skilled health personnel, stillbirth, postpartum haemorrhage, or maternal near-miss.

#### Antenatal care coverage

In Gullo et al. [26], the intervention did not show a significant effect on ANC coverage. While the difference-in-differences estimate showed a 4% increase in the number of women receiving ANC coverage in the intervention versus the control villages, this was not statistically significant and the estimate lacked precision (β = 0.04; 95% CI = -0.11, 0.18, *P* = 0.610).

#### Adolescent/youth pregnancy

Adolescent birth was not directly measured in any of the included studies but where a study provided data that enabled us to calculate as one of our outcomes, we used the authors’ data; instead of adolescent birth we considered adolescent/youth pregnancy along the pathway to adolescent (or youth) birth and thus examined the impact of interventions on pregnancy (among adolescents/youth). The studies by Cowan et al. [27], Ross et al. [28], and Jewkes et al. [29] reported pregnancy among adolescent or youth participants.

In Cowan et al. [27], we used the outcome “Currently pregnant”, measured at follow-up point of 36 months. We calculated currently pregnant youth (age 15-24 years) versus adolescent pregnancy (age 15-19 years) since we could not ascertain disaggregated ages of those currently pregnant. In Ross et al. [28], we used "Reported pregnancy during follow-up" as a proxy for adolescent/youth pregnancy. These data, measured at the 36-month follow-up, included participants from Years 4-6 of secondary school, i.e., young women who were 17 to >19 years old at the final survey. Jewkes et al. [29] assessed an outcome “unwanted pregnancy”. The authors defined this outcome as the participant (at time of study interview, either 12 or 24 months) was pregnant but wanted to become pregnant “later” or “not at all”. We calculated this as youth (unwanted) pregnancy (versus adolescent "unwanted” pregnancy since we could not ascertain disaggregated ages of participants currently with an "unwanted pregnancy" (at 24-month follow-up).

In Cowan et al. [27] there was no difference in odds of pregnancy between the two arms of the trial. However, there was a significant reduction in overall “reported pregnancies” among women. The prevalence of reported pregnancies among those in the intervention was 95/1237 (7.7%) and in the comparison group was 109/1349 (8.1%). The aOR was 0.92 (95% CI = 0.7, 1.19). Crude ORs were adjusted a priori for confounders of age, marital status, education, and strata used for randomisation, and any other potential confounders showing imbalance between intervention and control arms of the study.

In Ross et al. [28] overall, this study showed no significant difference in the prevalence of adolescent/youth pregnancy between trial arms. The prevalence of adolescent pregnancy among those in the intervention group was 489/1448 (46.9%) and in the comparison group was 489/1492 (45.5%). The aOR was 1.03 (95% CI = 0.89, 1.20). The investigators adjusted for school year (age group), marital status, ethnic group (“tribe”), lifetime number of partners, and stratum.

In Jewkes et al. [29] there was no evidence of the intervention having a protective effect on pregnancy (or any other secondary biological outcomes). It appears that the outcome of unwanted pregnancy was not in the intended direction, and that there was a "suggestion of more unwanted pregnancies at 24 months" in the intervention arm. The prevalence of pregnancy at 12 months for participants receiving the intervention was 78/537 (14.5%) versus 67/518 (12.9%) for those in the control group. The aOR was 1.14 (95% CI = 0.78, 1.67) [29]. The prevalence of pregnancy at 24 months in the intervention group was 75/521 (14.4%) versus 62/534 (11.6%) in the control group. The aOR of 1.45 (95% CI = 0.92, 2.28) [29]. ORs were adjusted for sex, age, baseline cluster prevalence of human immunodeficiency virus (HIV) or herpes simplex virus type 2 (HSV-2), and stratum [29].

### Results of syntheses

**Figure 3:**
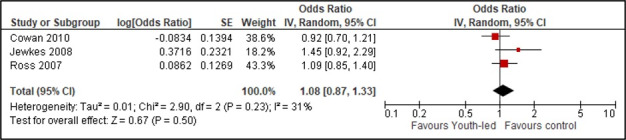
Forest plot of pooled odds ratio for adolescent/youth pregnancy. Source: [34].

We pooled data on adolescent/youth pregnancy from three studies (**Figure 3**). Youth-led interventions did not reduce the odds of adolescent/youth pregnancy (aOR = 1.08; 95% CI = 0.87, 1.33, *I*^2^ = 31%; three studies, low certainty of evidence). We had insufficient data to conduct any subgroup or sensitivity analysis.

### Reporting biases

We did not identify reporting biases based on time lag, duplicate publication, citation, or outcome reporting biases. We had insufficient studies to assess publication bias.

### Certainty of evidence

**Figure 4:**
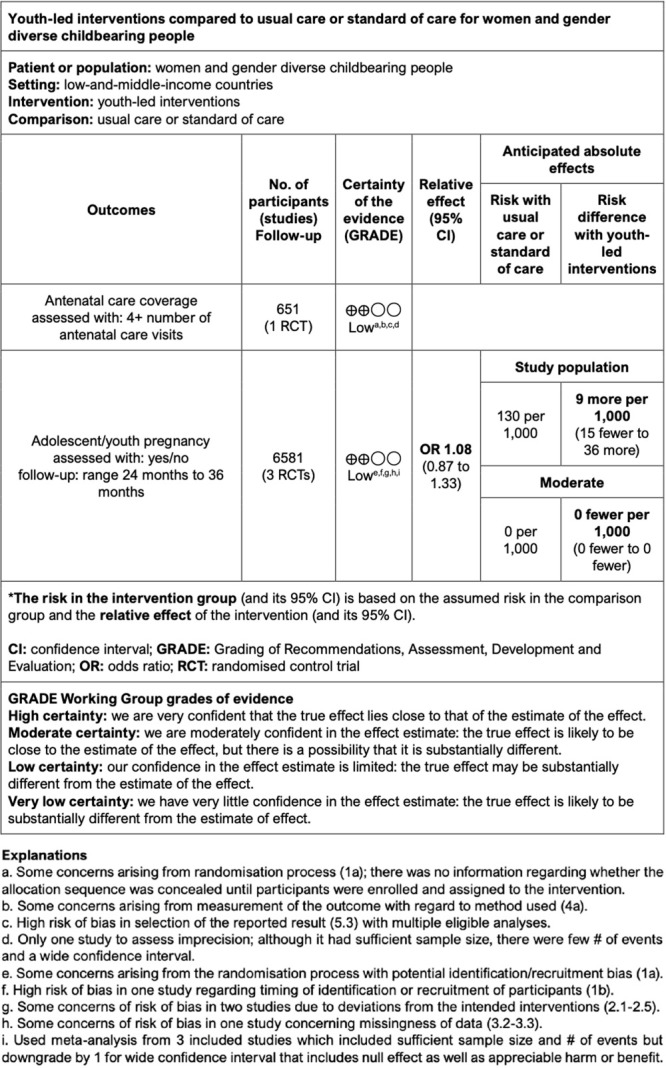
GRADE Summary of findings. Source: [23].

We included the outcomes of ANC coverage, and adolescent/youth pregnancy in the GRADE evidence profile. We assessed the certainty of the evidence as low. Our confidence in the effect estimate is limited since the true effect may be substantially different from the estimate of the effect. For both outcomes, we found serious risk of bias and imprecision in the included CRTs. We have reported a full description of our risk of bias judgement in the GRADE SoF table with footnotes explaining judgements (**Figure 4**).

## DISCUSSION

### Interpretation of results

In this systematic review of youth-led interventions for maternal and neonatal outcomes in LMICs, we included four trials, conducted in four African countries. One study reported no significant effect of a youth- led intervention on ANC coverage. Three studies showed no effect of youth-led interventions on adolescent/youth pregnancy. The certainty of the evidence for both outcomes was low.

Our review generated evidence on the impact of youth-led interventions in LMICs applied to people of childbearing age. Gullo et al. [26] speak to the importance of generating locally-relevant solutions, of using local level social accountability approaches and also the importance of youth involvement in reproductive health issues who can be a part of driving change in their communities. Process evaluation of youth involvement in the trial conducted by Cowan et al. showed that trained and motivated young people were an inspiration to the young people in the communities where they worked. An appreciation of the dynamic contexts of the communities where these studies took place is necessary to understand the potential impact on the implementation of the trials. For example, Cowan et al.’s trial was impacted by population mobility (i.e., a high rate of outmigration from the communities) and the fact that the intervention had become increasingly community-based [27]. The trialists consequently altered the design of their trial which affected their ability to detect intervention effectiveness. This aligns with a systematic review of interventions that integrate perinatal mental health care into routine maternal care in LMICs; and provides insights for overcoming barriers to designing and implementing interventions into routine care in LMICs [35]. Prom et al. [35] suggest the need for intervention adaptation to local healthcare settings and leveraging of locally available resources such as using peer mentors, which could be youth.

Complex interventions that involve behaviour change for adolescent or youth pregnancy prevention, for example, might require interventions that are more long-term or that require longer exposure [27,28]. A systematic review of potential interventions to improve adolescent sexual and reproductive health found that sexual and reproductive health education, counselling, and contraceptive availability are effective in decreasing adolescent pregnancy [36]. However, the authors were unable to conduct subgroup analyses for the effectiveness of interventions in high-income countries and LMICs because of limited studies from LMIC settings [36].

Cowan et al. [27] suggest the importance of finding ways to educate and convince policy makers, decision makers and other stakeholders that interventions targeting young people such as condom promotion, do no harm and do not increase risk-taking behaviours and would be key to successful scale-up of effective interventions.

Adverse events from the trials were not reported except in the trial by Jewkes et al. [29]. In this study the authors reported the deaths of 19 participants during the main study and pilot study. All deaths were investigated- none was found to be linked to activities of the study, and no other serious adverse events occurred.

### Limitations of evidence

Our study was limited by heterogeneity across the included studies as it pertained to participant characteristics, types of intervention, comparators, follow-up period, etc. We were only able to statistically pool results for one outcome. Risk of bias could only be assessed for the outcomes of antenatal care coverage and adolescent/youth pregnancy. In the studies by Gullo et al. [26] and by Jewkes et al. [29], there were concerns of overall high risk of bias across various domains for the respective outcomes of antenatal care coverage, and adolescent/youth pregnancy. We were unable to give an overall risk of bias per study.

### Limitations of review process

Despite our extensive search we may have missed (or possibly discarded) some eligible trials. Due to paucity of data, we were not able to conduct any subgroup analysis.

### Future directions

More research is needed in which youth are central to the research process to better understand how their engagement may be most effective in impacting community health. Pragmatic realist randomised trials that involve youth in a sustained way from conceptualisation, through planning and implementation may lead to evidence that shows both favourable process and outcome measures. Researchers and policy makers should incorporate youth as valuable and relevant community members where issues of health and well-being are being examined.

### Registration and protocol

We did not register this systematic review, but its protocol is available on PROSPERO (CRD42021288798). There were several deviations from our published protocol. We did not include Google because of its limitations as a searchable database. We did not explicitly reach out to key stakeholders/experts for additional sources of literature. But we did contact different authors about their studies and considered suggestions for additional sources from this paper’s co-authors. With respect to deviations in outcomes, we chose to report adolescent/youth pregnancy as a secondary outcome instead of Adolescent Birth rate. This deviation was based on the rationale that adolescent or youth pregnancy is along the continuum to adolescent or youth birth, and that we could expect that most pregnancies would lead to birth. We made a post hoc decision to collect information as to which WHO region the interventions occurred to provide more global-regional context.

## CONCLUSIONS

To the best of our knowledge, this is the first systematic review and meta-analysis that has examined the effects of community level **youth-led interventions** on maternal and neonatal health and well-being in LMICs. The results from our study do not suggest an improvement in maternal outcomes but this is based on a very small number of studies. More studies are required to make more precise conclusions. These studies should include outcomes of interest such as maternal death, neonatal death, postpartum hemorrhage, maternal near-miss, adolescent birth rate, and births attended by skilled health personnel.

## Additional material:


Online Supplementary Document

## References

[1] United Nations. Least Developed Countries (LDCs). 2023. Available: https://www.un.org/development/desa/dpad/least-developed-country-category.html. Accessed: 26 June 2023.

[2] World Health Organization. Trends in maternal mortality 2000 to 2017: estimates by WHO, UNICEF, UNFPA, World Bank Group and the United Nations Population Division. Geneva: World Health Organization; 2019.

[3] United Nations Chidren’s Fund. Levels and trends in child mortality: report 2020. New York: UNICEF; 2020.

[4] World Health Organization. Targets and strategies for ending preventable maternal mortality: consensus statement. Geneva: World Health Organization; 2014.

[5] United Nations. Definition of youth. 2019. Available: http://undesadspd.org/Youth.aspx. Accessed: 11 June 2019.

[6] Santhya KG Jejeebhoy SJ Sexual and reproductive health and rights of adolescent girls: evidence from low- and middle-income countries. Glob Public Health. 2015;10:189-221. 10.1080/17441692.2014.986169 25554828 PMC4318087

[7] Wood L Hendricks F A participatory action research approach to developing youth-friendly strategies for the prevention of teenage pregnancy. Educ Action Res. 2017;25:103-18. 10.1080/09650792.2016.1169198

[8] Alvarado G, Skinner M, Plaut D, Moss C, Kapungu C, Reavley N. A systematic review of positive youth development programs in low-and middle-income countries. Washington, DC: YouthPower Learning, Making Cents International; 2017. 10.1016/j.jadohealth.2019.01.024 31010725

[9] Yousafzai AK Rasheed MA Rizvi A Shaheen F Ponguta LA Reyes CR Effectiveness of a youth-led early childhood care and education programme in rural Pakistan: A cluster-randomised controlled trial. PLoS One. 2018;13:e0208335. 10.1371/journal.pone.0208335 30566498 PMC6300208

[10] Denison JA Burke VM Miti S Nonyane BAS Frimpong C Merrill KG Project YES! Youth Engaging for Success: A randomized controlled trial assessing the impact of a clinic-based peer mentoring program on viral suppression, adherence and internalized stigma among HIV-positive youth (15-24 years) in Ndola, Zambia. PLoS One. 2020;15:e0230703. 10.1371/journal.pone.0230703 32240186 PMC7117673

[11] Mannell J Willan S Shahmanesh M Seeley J Sherr L Gibbs A Why interventions to prevent intimate partner violence and HIV have failed young women in southern Africa. J Int AIDS Soc. 2019;22:e25380. 10.1002/jia2.25380 31441229 PMC6706780

[12] MacDonald T Gentles A Liu R Stevens-Uninsky M Anni NS Rehman N Community Level Youth-Led Interventions to Improve Maternal-Neonatal Outcomes in Low- and Middle-Income Countries: Protocol for a Systematic Review. Int J Reprod Med. 2022;2022:9580986. 10.1155/2022/9580986 35668840 PMC9167013

[13] McGowan J, Sampson M, Salzwedel D, Cogo E, Foerster V, Lefebvre C. PRESS Peer Review of Electronic Search Strategies:2015 Guideline Explanation and Elaboration (PRESS E&E). Ottawa: CADTH; 2016. 10.1016/j.jclinepi.2016.01.021 27005575

[14] Clark J Glasziou P Del Mar C Bannach-Brown A Stehlik P Scott AM A full systematic review was completed in 2 weeks using automation tools: a case study. J Clin Epidemiol. 2020;121:81-90. 10.1016/j.jclinepi.2020.01.008 32004673

[15] The EndNote Team. EndNote. EndNote X9 ed. Philadelphia, PA: Clarivate Analytics; 2013. Available: https://endnote.com/?language=en. Accessed: 5 December 2023.

[16] Distiller SR. 2.35. Introduction. 2021. Available: https://www.distillersr.com/. Accessed: 3 December 2023.

[17] Landis JR Koch GG The measurement of observer agreement for categorical data. Biometrics. 1977;33:159-74. 10.2307/2529310 843571

[18] The Cochrane Collaboration. Review Manager (RevMan). 5.4.1 ed: The Cochrane Collaboration. 2020. Available: revman.cochrane.org. Accessed: 3 December 2023.

[19] Sterne JAC Savović J Page MJ Elbers RG Blencowe NS Boutron I RoB 2: a revised tool for assessing risk of bias in randomised trials. BMJ. 2019;366:l4898. 10.1136/bmj.l4898 31462531

[20] The Cochrane Collaboration. Cochrane Methods: RoB2 for cluster-randomized trials. 2021. Available: https://sites.google.com/site/riskofbiastool/welcome/rob-2-0-tool/rob-2-for-cluster-randomized-trials. Accessed: 17 September 2021.

[21] Mbuagbaw L Medley N Darzi AJ Richardson M Habiba Garga K Ongolo-Zogo P Health system and community level interventions for improving antenatal care coverage and health outcomes. Cochrane Database Syst Rev. 2015;2015:CD010994. 10.1002/14651858.CD010994.pub2 26621223 PMC4676908

[22] Cochrane Training. Cochrane Handbook for Systematic Reviews of Interventions version 6.4. UK: Cochrane Training; 2023.

[23] GRADEpro GDT. GRADEpro Guideline Development Tool Software. McMaster University and Evidence Prime. 2023. Available: https://www.gradepro.org/. Accessed: 3 December 2023.

[24] Schünemann H, Brożek J, Guyatt G, Oxman A, editors. GRADE handbook for grading quality of evidence and strength of recommendations. Canada: The GRADE Working Group; 2013.

[25] Haddaway NR Page MJ Pritchard CC McGuinness LA PRISMA2020: An R package and Shiny app for producing PRISMA 2020-compliant flow diagrams, with interactivity for optimised digital transparency and Open Synthesis. Campbell Syst Rev. 2022;18:e1230. 10.1002/cl2.1230 36911350 PMC8958186

[26] Gullo S Galavotti C Kuhlmann AS Msiska T Hastings P Marti CN Effects of a social accountability approach, CARE’s Community Score Card, on reproductive health-related outcomes in Malawi: A cluster-randomized controlled evaluation. PLoS One. 2017;12:e0171316. 10.1371/journal.pone.0171316 28187159 PMC5302808

[27] Cowan FM Pascoe SJS Langhaug LF Mavhu W Chidiya S Jaffar S The Regai Dzive Shiri project: results of a randomized trial of an HIV prevention intervention for youth. AIDS. 2010;24:2541-52. 10.1097/QAD.0b013e32833e77c9 20881473 PMC3058934

[28] Ross DA Changalucha J Obasi AI Todd J Plummer ML Cleophas-Mazige B Biological and behavioural impact of an adolescent sexual health intervention in Tanzania: a community-randomized trial. AIDS. 2007;21:1943-55. 10.1097/QAD.0b013e3282ed3cf5 17721102

[29] Jewkes R Nduna M Levin J Jama N Dunkle K Puren A Impact of Stepping Stones on incidence of HIV and HSV-2 and sexual behaviour in rural South Africa: cluster randomised controlled trial. BMJ. 2008;337:a506. 10.1136/bmj.a506 18687720 PMC2505093

[30] Cowan FM Pascoe SJS Langhaug LF Dirawo J Chidiya S Jaffar S The Regai Dzive Shiri Project: a cluster randomised controlled trial to determine the effectiveness of a multi-component community-based HIV prevention intervention for rural youth in Zimbabwe - study design and baseline results. Trop Med Int Health. 2008;13:1235-44. 10.1111/j.1365-3156.2008.02137.x 18778329

[31] Hoffmann TC Glasziou PP Boutron I Milne R Perera R Moher D Better reporting of interventions: template for intervention description and replication (TIDieR) checklist and guide. BMJ. 2014;348:g1687. 10.1136/bmj.g1687 24609605

[32] Page MJ Moher D Bossuyt PM Boutron I Hoffmann TC Mulrow CD PRISMA 2020 explanation and elaboration: updated guidance and exemplars for reporting systematic reviews. BMJ. 2021;372:n160. 10.1136/bmj.n160 33781993 PMC8005925

[33] Page MJ McKenzie JE Bossuyt PM Boutron I Hoffmann TC Mulrow CD The PRISMA 2020 statement: an updated guideline for reporting systematic reviews. BMJ. 2021;372:n71. 10.1136/bmj.n71 33782057 PMC8005924

[34] McGuinness LA Higgins JPT Risk-of-bias VISualization (robvis): An R package and Shiny web app for visualizing risk-of-bias assessments. Res Synth Methods. 2021;12:55-61. 10.1002/jrsm.1411 32336025

[35] Prom MC Denduluri A Philpotts LL Rondon MB Borba CPC Gelaye B A systematic review of interventions that integrate perinatal mental health care into routine maternal care in low- and middle-income countries. Front Psychiatry. 2022;13:859341. 10.3389/fpsyt.2022.859341 35360136 PMC8964099

[36] Salam RA Faqqah A Sajjad N Lassi ZS Das JK Kaufman M Improving adolescent sexual and reproductive health: A systematic review of potential interventions. J Adolesc Health. 2016;59:S11-28. 10.1016/j.jadohealth.2016.05.022 27664592 PMC5026684

