# Supplementary material for: Community level youth-led interventions to improve maternal-neonatal outcomes in low- and middle-income countries: A systematic review of randomised trials
Source: J Glob Health. 2023 Dec 27;13:04168. doi: 10.7189/jogh.13.04168 (PMC10750450; doi:10.7189/jogh.13.04168)
Supplement: Online Supplementary Document [file jogh-13-04168-s001.pdf]

Online Supplementary Document

Table of Contents

Item	Description of item	Page
1.	Appendix S1: Definitions of Primary and Secondary Outcomes	2
2.	Appendix S2: Search Strategies	3
3.	Appendix S3: Data Items	32
4.	Appendix S4: Details of Four Included Studies	33
5.	Table S1: TIDieR Intervention Summary	36
6.	Table S2: PRIMSA 2020 for Abstracts Checklist	39
7.	Table S3: PRISMA 2020 Checklist	40
8.	References	43

Appendix S1
Definitions of Primary and Secondary Outcomes

Primary outcomes:

- Maternal deaths (i.e. pregnancy-related deaths): proportion of pregnant women/people who die during pregnancy or within 42 days of termination of pregnancy, irrespective of cause (obstetric, non-obstetric, accidental or incidental) [1].
- Neonatal deaths: proportion of newborn infants who die in the first 28 days after birth [2].

Secondary outcomes:

- Antenatal Care Coverage (ANC): proportion of pregnant women/people who attend at least four antenatal care visits during pregnancy [3].
- Births attended by skilled health personnel: proportion of pregnant women/people giving birth in health facilities.
- Adolescent birth rate: proportion of young women/people aged 15-19 years giving birth.
- Stillbirth: proportion of infants who die after 28 weeks of pregnancy, or before or during birth [4].
- Postpartum Hemorrhage (PPH): proportion of women/people who suffered from blood loss of 500 ml or more within 24 hours after birth [5].
- Maternal near-miss: proportion of women/people "who nearly died but survived a complication that occurred during pregnancy, childbirth or within 42 days of termination of pregnancy" [6].

Appendix S2
Search Strategies
This file contains search strategies of databases and registers (pgs 3-28), websites (pgs 28-30), organizations (pg 30), and grey literature (pgs 30-31) for this systematic review.

DATABASES AND REGISTERS
MEDLINE
Database: OVID Medline Epub Ahead of Print, In-Process & Other Non-Indexed Citations, Ovid MEDLINE(R) Daily and Ovid MEDLINE(R) 1946 to Present
Search date: Jan 21, 2022
----
1 (afghanistan or albania or algeria or american samoa or angola or "antigua and barbuda" or antigua or barbuda or argentina or armenia or armenian or aruba or azerbaijan or bahrain or bangladesh or barbados or republic of belarus or belarus or byelarus or belorussia or byelorussian or belize or british honduras or benin or dahomey or bhutan or bolivia or "bosnia and herzegovina" or bosnia or herzegovina or botswana or bechuanaland or brazil or brasil or bulgaria or burkina faso or burkina fasso or upper volta or burundi or urundi or cabo verde or cape verde or cambodia or kampuchea or khmer republic or cameroon or cameron or cameroun or central african republic or ubangi shari or chad or chile or china or colombia or comoros or comoro islands or iles comores or mayotte or democratic republic of the congo or democratic republic congo or congo or zaire or costa rica or "cote d'ivoire" or "cote d' ivoire" or cote divoire or cote d ivoire or ivory coast or croatia or cuba or cyprus or czech republic or czechoslovakia or djibouti or french somaliland or dominica or dominican republic or ecuador or egypt or united arab republic or el salvador or equatorial guinea or spanish guinea or eritrea or estonia or eswatini or swaziland or ethiopia or fiji or gabon or gabonese republic or gambia or "georgia (republic)" or georgian or ghana or gold coast or gibraltar or greece or grenada or guam or guatemala or guinea or guinea bissau or guyana or british guiana or haiti or hispaniola or honduras or hungary or india or indonesia or timor or iran or iraq or isle of man or jamaica or jordan or kazakhstan or kazakh or kenya or "democratic people's republic of korea" or republic of korea or north korea or south korea or korea or kosovo or kyrgyzstan or kirghizia or kirgizstan or kyrgyz republic or kirghiz or laos or lao pdr or "lao people's democratic republic" or latvia or lebanon or lebanese republic or lesotho or basutoland or liberia or libya or libyan arab jamahiriya or lithuania or macau or macao or republic of north macedonia or macedonia or madagascar or malagasy republic or malawi or nyasaland or malaysia or malay federation or malaya federation or maldives or indian ocean islands or indian ocean or mali or malta or micronesia or federated states of micronesia or kiribati or marshall islands or nauru or northern mariana islands or palau or tuvalu or mauritania or mauritius or mexico or moldova or moldovian or mongolia or montenegro or morocco or ifni or mozambique or portuguese east africa or myanmar or burma or namibia or nepal or netherlands antilles or nicaragua or niger or nigeria or oman or muscat or pakistan or panama or papua new guinea or new guinea or paraguay or peru or philippines or philipines or phillipines or phillippines or poland or "polish people's republic" or portugal or portuguese republic or puerto rico or romania or russia or russian federation or ussr or soviet union or union of soviet socialist republics or rwanda or ruanda or samoa or pacific islands or polynesia or samoan islands or navigator island or navigator islands or "sao tome and principe" or saudi arabia or senegal or serbia or seychelles or sierra leone or slovakia or slovak republic or slovenia or melanesia or solomon island or solomon islands or norfolk island or norfolk islands or somalia or south africa or south sudan or sri lanka or ceylon or "saint kitts and nevis" or "st. kitts and nevis" or saint lucia or "st. lucia" or "saint vincent and the grenadines" or saint vincent or "st. vincent" or grenadines or sudan or suriname or surinam or dutch guiana or netherlands guiana or syria or syrian arab republic or tajikistan or tadjikistan or tadzhikistan or tadzhik or tanzania or tanganyika or thailand or siam or timor leste or east timor or togo or togolese republic or tonga or "trinidad and tobago" or trinidad or tobago or tunisia or turkey or turkmenistan or turkmen or uganda or ukraine or uruguay or uzbekistan or uzbek or vanuatu or new hebrides or venezuela or vietnam or viet nam or middle east or west bank or gaza or palestine or yemen or yugoslavia or zambia or zimbabwe or northern rhodesia or global south or africa south of the sahara or sub-saharan africa or subsaharan africa or africa, central or central africa or africa, northern or north africa or northern africa or magreb or maghrib or sahara or africa, southern or southern africa or africa, eastern or east africa or eastern africa or africa, western or west africa or western africa or

west indies or indian ocean islands or caribbean or central america or latin america or "south and central america" or south america or asia, central or central asia or asia, northern or north asia or northern asia or asia, southeastern or southeastern asia or south eastern asia or southeast asia or south east asia or asia, western or western asia or europe, eastern or east europe or eastern europe or developing country or developing countries or developing nation? or developing population? or developing world or less developed countr* or less developed nation? or less developed population? or less developed world or lesser developed countr* or lesser developed nation? or lesser developed population? or lesser developed world or under developed countr* or under developed nation? or under developed population? or under developed world or underdeveloped countr* or underdeveloped nation? or underdeveloped population? or underdeveloped world or middle income countr* or middle income nation? or middle income population? or low income countr* or low income nation? or low income population? or lower income countr* or lower income nation? or lower income population? or underserved countr* or underserved nation? or underserved population? or underserved world or under served countr* or under served nation? or under served population? or under served world or deprived countr* or deprived nation? or deprived population? or deprived world or poor countr* or poor nation? or poor population? or poor world or poorer countr* or poorer nation? or poorer population? or poorer world or developing econom* or less developed econom* or lesser developed econom* or under developed econom* or underdeveloped econom* or middle income econom* or low income econom* or lower income econom* or low gdp or low gnp or low gross domestic or low gross national or lower gdp or lower gnp or lower gross domestic or lower gross national or lmic or lmics or third world or lami countr* or transitional countr* or emerging economies or emerging nation?).ti,ab,sh,kf. (2164431)
2 exp peer group/ (23263)
3 ((youth* or young* or highschool* or high school* or student* or adolescent* or peer* or member* or kid* or teen* or coming of age) adj3 (leader* or design* or facilitat* or develop* or initiat* or coordinat* or serve* or intervention* or initiative* or project* or program* or campaign* or promotion*)).ti,ab. (104751)
4 2 or 3 (123097)
5 1 and 4 (15077)
6 Maternal Health/ (2040)
7 exp Pregnancy/ (952281)
8 exp postpartum period/ (70641)
9 Peripartum Period/ (1525)
10 pregnan*.ti,ab. (547075)
11 postpartum.ti,ab. (60005)
12 Peripartum.ti,ab. (5479)
13 Maternal Health.ti,ab. (7219)
14 maternal wellbeing.ti,ab. (108)
15 maternal well-being.ti,ab. (374)
16 exp Infant, Newborn/ (645037)
17 neonat*.mp. (316777)
18 or/6-17 (1669732)
19 5 and 18 (1614)

MEDLINE (update in 2023)
Database: OVID Medline Epub Ahead of Print, In-Process & Other Non-Indexed Citations, Ovid MEDLINE(R) Daily and Ovid MEDLINE(R) 1946 to Present
Search date: Jan 30, 2023
----
1 (afghanistan or albania or algeria or american samoa or angola or "antigua and barbuda" or antigua or barbuda or argentina or armenia or armenian or aruba or azerbaijan or bahrain or bangladesh or barbados or republic of belarus or belarus or byelarus or belorussia or byelorussian or belize or british honduras or benin or dahomey or bhutan or bolivia or "bosnia and herzegovina" or bosnia or herzegovina or botswana or bechuanaland or brazil or brasil or bulgaria or burkina faso or burkina fasso or upper volta or burundi or urundi or cabo verde or cape verde or cambodia or kampuchea or khmer republic or cameroon or cameron

or cameroun or central african republic or ubangi shari or chad or chile or china or colombia or comoros or comoro islands or iles comores or mayotte or democratic republic of the congo or democratic republic congo or congo or zaire or costa rica or "cote d'ivoire" or "cote d' ivoire" or cote divoire or cote d ivoire or ivory coast or croatia or cuba or cyprus or czech republic or czechoslovakia or djibouti or french somaliland or dominica or dominican republic or ecuador or egypt or united arab republic or el salvador or equatorial guinea or spanish guinea or eritrea or estonia or eswatini or swaziland or ethiopia or fiji or gabon or gabonese republic or gambia or "georgia (republic)" or georgian or ghana or gold coast or gibraltar or greece or grenada or guam or guatemala or guinea or guinea bissau or guyana or british guiana or haiti or hispaniola or honduras or hungary or india or indonesia or timor or iran or iraq or isle of man or jamaica or jordan or kazakhstan or kazakh or kenya or "democratic people's republic of korea" or republic of korea or north korea or south korea or korea or kosovo or kyrgyzstan or kirghizia or kirgizstan or kyrgyz republic or kirghiz or laos or lao pdr or "lao people's democratic republic" or latvia or lebanon or lebanese republic or lesotho or basutoland or liberia or libya or libyan arab jamahiriya or lithuania or macau or macao or republic of north macedonia or macedonia or madagascar or malagasy republic or malawi or nyasaland or malaysia or malay federation or malaya federation or maldives or indian ocean islands or indian ocean or mali or malta or micronesia or federated states of micronesia or kiribati or marshall islands or nauru or northern mariana islands or palau or tuvalu or mauritania or mauritius or mexico or moldova or moldovian or mongolia or montenegro or morocco or ifni or mozambique or portuguese east africa or myanmar or burma or namibia or nepal or netherlands antilles or nicaragua or niger or nigeria or oman or muscat or pakistan or panama or papua new guinea or new guinea or paraguay or peru or philippines or philipines or phillipines or phillippines or poland or "polish people's republic" or portugal or portuguese republic or puerto rico or romania or russia or russian federation or ussr or soviet union or union of soviet socialist republics or rwanda or ruanda or samoa or pacific islands or polynesia or samoan islands or navigator island or navigator islands or "sao tome and principe" or saudi arabia or senegal or serbia or seychelles or sierra leone or slovakia or slovak republic or slovenia or melanesia or solomon island or solomon islands or norfolk island or norfolk islands or somalia or south africa or south sudan or sri lanka or ceylon or "saint kitts and nevis" or "st. kitts and nevis" or saint lucia or "st. lucia" or "saint vincent and the grenadines" or saint vincent or "st. vincent" or grenadines or sudan or suriname or surinam or dutch guiana or netherlands guiana or syria or syrian arab republic or tajikistan or tadjikistan or tadzhikistan or tadzhik or tanzania or tanganyika or thailand or siam or timor leste or east timor or togo or togolese republic or tonga or "trinidad and tobago" or trinidad or tobago or tunisia or turkey or turkmenistan or turkmen or uganda or ukraine or uruguay or uzbekistan or uzbek or vanuatu or new hebrides or venezuela or vietnam or viet nam or middle east or west bank or gaza or palestine or yemen or yugoslavia or zambia or zimbabwe or northern rhodesia or global south or africa south of the sahara or sub-saharan africa or subsaharan africa or africa, central or central africa or africa, northern or north africa or northern africa or magreb or maghrib or sahara or africa, southern or southern africa or africa, eastern or east africa or eastern africa or africa, western or west africa or western africa or west indies or indian ocean islands or caribbean or central america or latin america or "south and central america" or south america or asia, central or central asia or asia, northern or north asia or northern asia or asia, southeastern or southeastern asia or south eastern asia or southeast asia or south east asia or asia, western or western asia or europe, eastern or east europe or eastern europe or developing country or developing countries or developing nation? or developing population? or developing world or less developed countr* or less developed nation? or less developed population? or less developed world or lesser developed countr* or lesser developed nation? or lesser developed population? or lesser developed world or under developed countr* or under developed nation? or under developed population? or under developed world or underdeveloped countr* or underdeveloped nation? or underdeveloped population? or underdeveloped world or middle income countr* or middle income nation? or middle income population? or low income countr* or low income nation? or low income population? or lower income countr* or lower income nation? or lower income population? or underserved countr* or underserved nation? or underserved population? or underserved world or under served countr* or under served nation? or under served population? or under served world or deprived countr* or deprived nation? or deprived population? or deprived world or poor countr* or poor nation? or poor population? or poor world or poorer countr* or poorer nation? or poorer population? or poorer world or developing econom* or less developed econom* or lesser developed econom* or under developed econom* or underdeveloped econom* or middle income

econom* or low income econom* or lower income econom* or low gdp or low gnp or low gross domestic or low gross national or lower gdp or lower gnp or lower gross domestic or lower gross national or lmic or lmics or third world or lami countr* or transitional countr* or emerging economies or emerging nation?).ti,ab,sh,kf. (2331687)
2 exp peer group/ (24416)
3 ((youth* or young* or highschool* or high school* or student* or adolescent* or peer* or member* or kid* or teen* or coming of age) adj3 (leader* or design* or facilitat* or develop* or initiat* or coordinat* or serve* or intervention* or initiative* or project* or program* or campaign* or promotion*)).ti,ab. (115127)
4 2 or 3 (133821) 134262
5 1 and 4 (16978)
6 Maternal Health/ (2230)
7 exp Pregnancy/ (991450)
8 exp postpartum period/ (74175)
9 Peripartum Period/ (1689)
10 pregnan*.ti,ab. (577969)

11 postpartum.ti,ab. (65039)
12 Peripartum.ti,ab. (6029)
13 Maternal Health.ti,ab. (8125)
14 maternal wellbeing.ti,ab. (126)
15 maternal well-being.ti,ab. (412)
16 exp Infant, Newborn/ (665868)
17 neonat*.mp. (333041)
18 or/6-17 (1732744)
19 5 and 18 (1754)
20 limit 19 to ed=20220121-20230124 (111)
21 limit 19 to yr="2022 -Current" (142)
22 20 or 21 (152)

Embase
Database: Embase <1974 to 2022 January 20>
Search date: Jan 21, 2022
----
1 (afghanistan or albania or algeria or american samoa or angola or "antigua and barbuda" or antigua or barbuda or argentina or armenia or armenian or aruba or azerbaijan or bahrain or bangladesh or barbados or republic of belarus or belarus or byelarus or belorussia or byelorussian or belize or british honduras or benin or dahomey or bhutan or bolivia or "bosnia and herzegovina" or bosnia or herzegovina or botswana or bechuanaland or brazil or brasil or bulgaria or burkina faso or burkina fasso or upper volta or burundi or urundi or cabo verde or cape verde or cambodia or kampuchea or khmer republic or cameroon or cameron or cameroun or central african republic or ubangi shari or chad or chile or china or colombia or comoros or comoro islands or iles comores or mayotte or democratic republic of the congo or democratic republic congo or congo or zaire or costa rica or "cote d’ivoire" or "cote d’ ivoire" or cote divoire or cote d ivoire or ivory coast or croatia or cuba or cyprus or czech republic or czechoslovakia or djibouti or french somaliland or dominica or dominican republic or ecuador or egypt or united arab republic or el salvador or equatorial guinea or spanish guinea or eritrea or estonia or eswatini or swaziland or ethiopia or fiji or gabon or gabonese republic or gambia or "georgia (republic)" or georgian or ghana or gold coast or gibraltar or greece or grenada or guam or guatemala or guinea or guinea bissau or guyana or british guiana or haiti or hispaniola or honduras or hungary or india or indonesia or timor or iran or iraq or isle of man or jamaica or jordan or kazakhstan or kazakh or kenya or "democratic people’s republic of korea" or republic of korea or north korea or south korea or korea or kosovo or kyrgyzstan or kirghizia or kirgizstan or kyrgyz republic or kirghiz or laos or lao pdr or "lao people's democratic republic" or latvia or lebanon or lebanese republic or lesotho or basutoland or liberia or libya or libyan arab jamahiriya or lithuania or macau or macao or republic of north macedonia or macedonia or madagascar or malagasy republic or malawi or nyasaland or malaysia

or malay federation or malaya federation or maldives or indian ocean islands or indian ocean or mali or malta or micronesia or federated states of micronesia or kiribati or marshall islands or nauru or northern mariana islands or palau or tuvalu or mauritania or mauritius or mexico or moldova or moldovian or mongolia or montenegro or "montenegro (republic)" or morocco or ifni or mozambique or portuguese east africa or myanmar or burma or namibia or nepal or netherlands antilles or nicaragua or niger or nigeria or oman or muscat or pakistan or panama or papua new guinea or new guinea or paraguay or peru or philippines or philipines or phillipines or phillippines or poland or "polish people's republic" or portugal or portuguese republic or puerto rico or romania or russia or russian federation or ussr or soviet union or union of soviet socialist republics or rwanda or ruanda or samoa or pacific islands or polynesia or samoan islands or navigator island or navigator islands or "sao tome and principe" or saudi arabia or senegal or serbia or seychelles or sierra leone or slovakia or slovak republic or slovenia or melanesia or solomon island or solomon islands or norfolk island or norfolk islands or somalia or south africa or south sudan or sri lanka or ceylon or "saint kitts and nevis" or "st. kitts and nevis" or saint lucia or "st. lucia" or "saint vincent and the grenadines" or saint vincent or "st. vincent" or grenadines or sudan or suriname or surinam or dutch guiana or netherlands guiana or syria or syrian arab republic or tajikistan or tadjikistan or tadzhikistan or tadzhik or tanzania or tanganyika or thailand or siam or timor leste or east timor or togo or togolese republic or tonga or "trinidad and tobago" or trinidad or tobago or tunisia or "turkey (republic)" or turkey or turkmenistan or turkmen or uganda or ukraine or uruguay or uzbekistan or uzbek or vanuatu or new hebrides or venezuela or vietnam or viet nam or middle east or west bank or gaza or palestine or yemen or yugoslavia or zambia or zimbabwe or northern rhodesia or global south or africa south of the sahara or "sub saharan africa" or subsaharan africa or africa, central or central africa or africa, northern or north africa or northern africa or magreb or maghrib or sahara or africa, southern or southern africa or africa, eastern or east africa or eastern africa or africa, western or west africa or western africa or west indies or indian ocean islands or caribbean region or caribbean islands or caribbean or central america or latin america or "south and central america" or south america or asia, central or central asia or asia, northern or north asia or northern asia or asia, southeastern or southeastern asia or south eastern asia or southeast asia or south east asia or asia, western or western asia or europe, eastern or east europe or eastern europe or developing country or developing countries or developing nation? or developing population? or developing world or less developed countr* or less developed nation? or less developed population? or less developed world or lesser developed countr* or lesser developed nation? or lesser developed population? or lesser developed world or under developed countr* or under developed nation? or under developed population? or under developed world or underdeveloped countr* or underdeveloped nation? or underdeveloped population? or underdeveloped world or middle income countr* or middle income nation? or middle income population? or low income countr* or low income nation? or low income population? or lower income countr* or lower income nation? or lower income population? or underserved countr* or underserved nation? or underserved population? or underserved world or under served countr* or under served nation? or under served population? or under served world or deprived countr* or deprived nation? or deprived population? or deprived world or poor countr* or poor nation? or poor population? or poor world or poorer countr* or poorer nation? or poorer population? or poorer world or developing econom* or less developed econom* or lesser developed econom* or under developed econom* or underdeveloped econom* or middle income econom* or low income econom* or lower income econom* or low gdp or low gnp or low gross domestic or low gross national or lower gdp or lower gnp or lower gross domestic or lower gross national or lmic or lmics or third world or lami countr* or transitional countr* or emerging economies or emerging nation?).ti,ab,sh,kw. (2436147)

2 exp peer group/ (27013)

3 ((youth* or young* or highschool* or high school* or student* or adolescent* or peer* or member* or kid* or teen* or coming of age) adj3 (leader* or design* or facilitat* or develop* or initiat* or coordinat* or serve* or intervention* or initiative* or project* or program* or campaign* or promotion*)).ti,ab. (140496)

4 2 or 3 (161881)

5 1 and 4 (19295)

6 exp maternal welfare/ (15321)

7 maternal health.mp. (11484)

8 6 or 7 (22397)

9 exp pregnancy/ (709226)
10 pregnancy.mp. (926103)
11 exp puerperium/ (71435)
12 puerperium.mp. (45129)
13 exp perinatal period/ (37763)
14 peripartum period.mp. (1580)
15 pregnan*.ti,ab. (693394)
16 postpartum.ti,ab. (77655)
17 Peripartum.ti,ab. (8112)
18 Maternal Health.ti,ab. (8242)
19 maternal wellbeing.ti,ab. (163)
20 maternal well-being.ti,ab. (452)
21 exp infant/ (1054018)
22 neonat*.mp. (391159)
23 6 or 7 or 8 or 9 or 10 or 11 or 12 or 13 or 14 or 15 or 16 or 17 or 18 or 19 or 20 or 21 or 22 (2063232)
24 5 and 23 (2247)

Embase (update in 2023)
Database: Embase <1974 to 2023 January 23>
Search date: Jan 24, 2023
----
1 (afghanistan or albania or algeria or american samoa or angola or "antigua and barbuda" or antigua or barbuda or argentina or armenia or armenian or aruba or azerbaijan or bahrain or bangladesh or barbados or republic of belarus or belarus or byelarus or belorussia or byelorussian or belize or british honduras or benin or dahomey or bhutan or bolivia or "bosnia and herzegovina" or bosnia or herzegovina or botswana or bechuanaland or brazil or brasil or bulgaria or burkina faso or burkina fasso or upper volta or burundi or urundi or cabo verde or cape verde or cambodia or kampuchea or khmer republic or cameroon or cameron or cameroun or central african republic or ubangi shari or chad or chile or china or colombia or comoros or comoro islands or iles comores or mayotte or democratic republic of the congo or democratic republic congo or congo or zaire or costa rica or "cote d'ivoire" or "cote d' ivoire" or cote divoire or cote d ivoire or ivory coast or croatia or cuba or cyprus or czech republic or czechoslovakia or djibouti or french somaliland or dominica or dominican republic or ecuador or egypt or united arab republic or el salvador or equatorial guinea or spanish guinea or eritrea or estonia or eswatini or swaziland or ethiopia or fiji or gabon or gabonese republic or gambia or "georgia (republic)" or georgian or ghana or gold coast or gibraltar or greece or grenada or guam or guatemala or guinea or guinea bissau or guyana or british guiana or haiti or hispaniola or honduras or hungary or india or indonesia or timor or iran or iraq or isle of man or jamaica or jordan or kazakhstan or kazakh or kenya or "democratic people's republic of korea" or republic of korea or north korea or south korea or korea or kosovo or kyrgyzstan or kirghizia or kirgizstan or kyrgyz republic or kirghiz or laos or lao pdr or "lao people's democratic republic" or latvia or lebanon or lebanese republic or lesotho or basutoland or liberia or libya or libyan arab jamahiriya or lithuania or macau or macao or republic of north macedonia or macedonia or madagascar or malagasy republic or malawi or nyasaland or malaysia or malay federation or malaya federation or maldives or indian ocean islands or indian ocean or mali or malta or micronesia or federated states of micronesia or kiribati or marshall islands or nauru or northern mariana islands or palau or tuvalu or mauritania or mauritius or mexico or moldova or moldovian or mongolia or montenegro or "montenegro (republic)" or morocco or ifni or mozambique or portuguese east africa or myanmar or burma or namibia or nepal or netherlands antilles or nicaragua or niger or nigeria or oman or muscat or pakistan or panama or papua new guinea or new guinea or paraguay or peru or philippines or philipines or phillipines or phillippines or poland or "polish people's republic" or portugal or portuguese republic or puerto rico or romania or russia or russian federation or ussr or soviet union or union of soviet socialist republics or rwanda or ruanda or samoa or pacific islands or polynesia or samoan islands or navigator island or navigator islands or "sao tome and principe" or saudi arabia or senegal or serbia or seychelles or sierra leone or slovakia or slovak republic or slovenia or melanesia or solomon island or solomon islands or norfolk island or norfolk islands or somalia or south africa or south sudan or sri lanka or

ceylon or "saint kitts and nevis" or "st. kitts and nevis" or saint lucia or "st. lucia" or "saint vincent and the grenadines" or saint vincent or "st. vincent" or grenadines or sudan or suriname or surinam or dutch guiana or netherlands guiana or syria or syrian arab republic or tajikistan or tadjikistan or tadzhikistan or tadzhik or tanzania or tanganyika or thailand or siam or timor leste or east timor or togo or togolese republic or tonga or "trinidad and tobago" or trinidad or tobago or tunisia or "turkey (republic)" or turkey or turkmenistan or turkmen or uganda or ukraine or uruguay or uzbekistan or uzbek or vanuatu or new hebrides or venezuela or vietnam or viet nam or middle east or west bank or gaza or palestine or yemen or yugoslavia or zambia or zimbabwe or northern rhodesia or global south or africa south of the sahara or "sub saharan africa" or subsaharan africa or africa, central or central africa or africa, northern or north africa or northern africa or magreb or maghrib or sahara or africa, southern or southern africa or africa, eastern or east africa or eastern africa or africa, western or west africa or western africa or west indies or indian ocean islands or caribbean region or caribbean islands or caribbean or central america or latin america or "south and central america" or south america or asia, central or central asia or asia, northern or north asia or northern asia or asia, southeastern or southeastern asia or south eastern asia or southeast asia or south east asia or asia, western or western asia or europe, eastern or east europe or eastern europe or developing country or developing countries or developing nation? or developing population? or developing world or less developed countr* or less developed nation? or less developed population? or less developed world or lesser developed countr* or lesser developed nation? or lesser developed population? or lesser developed world or under developed countr* or under developed nation? or under developed population? or under developed world or underdeveloped countr* or underdeveloped nation? or underdeveloped population? or underdeveloped world or middle income countr* or middle income nation? or middle income population? or low income countr* or low income nation? or low income population? or lower income countr* or lower income nation? or lower income population? or underserved countr* or underserved nation? or underserved population? or underserved world or under served countr* or under served nation? or under served population? or under served world or deprived countr* or deprived nation? or deprived population? or deprived world or poor countr* or poor nation? or poor population? or poor world or poorer countr* or poorer nation? or poorer population? or poorer world or developing econom* or less developed econom* or lesser developed econom* or under developed econom* or underdeveloped econom* or middle income econom* or low income econom* or lower income econom* or low gdp or low gnp or low gross domestic or low gross national or lower gdp or lower gnp or lower gross domestic or lower gross national or lmic or lmics or third world or lami countr* or transitional countr* or emerging economies or emerging nation?).ti,ab,sh,kw. (2638486)

2 exp peer group/ (29156)

3 ((youth* or young* or highschool* or high school* or student* or adolescent* or peer* or member* or kid* or teen* or coming of age) adj3 (leader* or design* or facilitat* or develop* or initiat* or coordinat* or serve* or intervention* or initiative* or project* or program* or campaign* or promotion*)).ti,ab. (154542)

4 2 or 3 (177558)

5 1 and 4 (21475)

6 exp maternal welfare/ (16530)

7 maternal health.mp. (13126)

8 6 or 7 (24481)

9 exp pregnancy/ (752896)

10 pregnancy.mp. (984226)

11 exp puerperium/ (77402)

12 puerperium.mp. (48580)

13 exp perinatal period/ (39956)

14 peripartum period.mp. (1761)

15 pregnan*.ti,ab. (743969)

16 postpartum.ti,ab. (85144)

17 Peripartum.ti,ab. (9005)

18 Maternal Health.ti,ab. (9288)

19 maternal wellbeing.ti,ab. (198)

20 maternal well-being.ti,ab. (484)

21 exp infant/ (1106603)
22 neonat*.mp. (416961)
23 6 or 7 or 8 or 9 or 10 or 11 or 12 or 13 or 14 or 15 or 16 or 17 or 18 or 19 or 20 or 21 or 22 (2176976)
24 5 and 23 (2449)
25 limit 24 to dc=20220121-20230124 (218)
26 limit 24 to yr="2022 -Current" (189)
27 25 or 26 (224)

CINAHL (update in 2023)
Database: CINAHL limiter: publication date 20220101-20231231
Search date: Jan 25, 2023

S1 TI (afghanistan or albania or algeria or american samoa or angola or "antigua and barbuda" or antigua or barbuda or argentina or armenia or armenian or aruba or azerbaijan or bahrain or bangladesh or barbados or republic of belarus or belarus or byelarus or belorussia or byelorussian or belize or british honduras or benin or dahomey or bhutan or bolivia or "bosnia and herzegovina" or bosnia or herzegovina or botswana or bechuanaland or brazil or brasil or bulgaria or burkina faso or burkina fasso or upper volta or burundi or urundi or cabo verde or cape verde or cambodia or kampuchea or khmer republic or cameroon or cameron or cameroun or central african republic or ubangi shari or chad or chile or china or colombia or comoros or comoro islands or iles comores or mayotte or democratic republic of the congo or democratic republic congo or congo or zaire or costa rica or "cote d’ivoire" or "cote d’ ivoire" or cote divoire or cote d ivoire or ivory coast or croatia or cuba or cyprus or czech republic or czechoslovakia or djibouti or french somaliland or dominica or dominican republic or ecuador or egypt or united arab republic or el salvador or equatorial guinea or spanish guinea or eritrea or estonia or eswatini or swaziland or ethiopia or fiji or gabon or gabonese republic or gambia or "georgia (republic)" or georgian or ghana or gold coast or gibraltar or greece or grenada or guam or guatemala or guinea or guinea bissau or guyana or british guiana or haiti or hispaniola or honduras or hungary or india or indonesia or timor or iran or iraq or isle of man or jamaica or jordan or kazakhstan or kazakh or kenya or "democratic people’s republic of korea" or republic of korea or north korea or south korea or korea or kosovo or kyrgyzstan or kirghizia or kirgizstan or kyrgyz republic or kirghiz or laos or lao pdr or "lao people's democratic republic" or latvia or lebanon or lebanese republic or lesotho or basutoland or liberia or libya or libyan arab jamahiriya or lithuania or macau or macao or republic of north macedonia or macedonia or madagascar or malagasy republic or malawi or nyasaland or malaysia or malay federation or malaya federation or maldives or indian ocean islands or indian ocean or mali or malta or micronesia or federated states of micronesia or kiribati or marshall islands or nauru or northern mariana islands or palau or tuvalu or mauritania or mauritius or mexico or moldova or moldovian or mongolia or montenegro or morocco or ifni or mozambique or portuguese east africa or myanmar or burma or namibia or nepal or netherlands antilles or nicaragua or niger or nigeria or oman or muscat or pakistan or panama or papua new guinea or new guinea or paraguay or peru or philippines or philipines or phillipines or phillippines or poland or "polish people's republic" or portugal or portuguese republic or puerto rico or romania or russia or russian federation or ussr or soviet union or union of soviet socialist republics or rwanda or ruanda or samoa or pacific islands or polynesia or samoan islands or navigator island or navigator islands or "sao tome and principe" or saudi arabia or senegal or serbia or seychelles or sierra leone or slovakia or slovak republic or slovenia or melanesia or solomon island or solomon islands or norfolk island or norfolk islands or somalia or south africa or south sudan or sri lanka or ceylon or "saint kitts and nevis" or "st. kitts and nevis" or saint lucia or "st. lucia" or "saint vincent and the grenadines" or saint vincent or "st. vincent" or grenadines or sudan or suriname or surinam or dutch guiana or netherlands guiana or syria or syrian arab republic or tajikistan or tadjikistan or tadzhikistan or tadzhik or tanzania or tanganyika or thailand or siam or timor leste or east timor or togo or togolese republic or tonga or "trinidad and tobago" or trinidad or tobago or tunisia or turkey or turkmenistan or turkmen or uganda or ukraine or uruguay or uzbekistan or uzbek or vanuatu or new hebrides or venezuela or vietnam or viet nam or middle east or west bank or gaza or palestine or yemen or yugoslavia or zambia or zimbabwe or northern rhodesia or global south or africa south of the sahara or sub-saharan africa or subsaharan africa or africa, central or central africa or africa, northern or north africa or northern africa or magreb or maghrib or sahara or africa, southern

or southern africa or africa, eastern or east africa or eastern africa or africa, western or west africa or western africa or west indies or indian ocean islands or caribbean or central america or latin america or "south and central america" or south america or asia, central or central asia or asia, northern or north asia or northern asia or asia, southeastern or southeastern asia or south eastern asia or southeast asia or south east asia or asia, western or western asia or europe, eastern or east europe or eastern europe or developing country or developing countries or developing nation? or developing population? or developing world or less developed countr* or less developed nation? or less developed population? or less developed world or lesser developed countr* or lesser developed nation? or lesser developed population? or lesser developed world or under developed countr* or under developed nation? or under developed population? or under developed world or underdeveloped countr* or underdeveloped nation? or underdeveloped population? or underdeveloped world or middle income countr* or middle income nation? or middle income population? or low income countr* or low income nation? or low income population? or lower income countr* or lower income nation? or lower income population? or underserved countr* or underserved nation? or underserved population? or underserved world or under served countr* or under served nation? or under served population? or under served world or deprived countr* or deprived nation? or deprived population? or deprived world or poor countr* or poor nation? or poor population? or poor world or poorer countr* or poorer nation? or poorer population? or poorer world or developing econom* or less developed econom* or lesser developed econom* or under developed econom* or underdeveloped econom* or middle income econom* or low income econom* or lower income econom* or low gdp or low gnp or low gross domestic or low gross national or lower gdp or lower gnp or lower gross domestic or lower gross national or lmic or lmics or third world or lami countr* or transitional countr* or emerging economies or emerging nation?)) OR AB ((afghanistan or albania or algeria or american samoa or angola or "antigua and barbuda" or antigua or barbuda or argentina or armenia or armenian or aruba or azerbaijan or bahrain or bangladesh or barbados or republic of belarus or belarus or byelarus or belorussia or byelorussian or belize or british honduras or benin or dahomey or bhutan or bolivia or "bosnia and herzegovina" or bosnia or herzegovina or botswana or bechuanaland or brazil or brasil or bulgaria or burkina faso or burkina fasso or upper volta or burundi or urundi or cabo verde or cape verde or cambodia or kampuchea or khmer republic or cameroon or cameron or cameroun or central african republic or ubangi shari or chad or chile or china or colombia or comoros or comoro islands or iles comores or mayotte or democratic republic of the congo or democratic republic congo or congo or zaire or costa rica or "cote d’ivoire" or "cote d’ ivoire" or cote divoire or cote d ivoire or ivory coast or croatia or cuba or cyprus or czech republic or czechoslovakia or djibouti or french somaliland or dominica or dominican republic or ecuador or egypt or united arab republic or el salvador or equatorial guinea or spanish guinea or eritrea or estonia or eswatini or swaziland or ethiopia or fiji or gabon or gabonese republic or gambia or "georgia (republic)" or georgian or ghana or gold coast or gibraltar or greece or grenada or guam or guatemala or guinea or guinea bissau or guyana or british guiana or haiti or hispaniola or honduras or hungary or india or indonesia or timor or iran or iraq or isle of man or jamaica or jordan or kazakhstan or kazakh or kenya or "democratic people’s republic of korea" or republic of korea or north korea or south korea or korea or kosovo or kyrgyzstan or kirghizia or kirgizstan or kyrgyz republic or kirghiz or laos or lao pdr or "lao people's democratic republic" or latvia or lebanon or lebanese republic or lesotho or basutoland or liberia or libya or libyan arab jamahiriya or lithuania or macau or macao or republic of north macedonia or macedonia or madagascar or malagasy republic or malawi or nyasaland or malaysia or malay federation or malaya federation or maldives or indian ocean islands or indian ocean or mali or malta or micronesia or federated states of micronesia or kiribati or marshall islands or nauru or northern mariana islands or palau or tuvalu or mauritania or mauritius or mexico or moldova or moldovian or mongolia or montenegro or morocco or ifni or mozambique or portuguese east africa or myanmar or burma or namibia or nepal or netherlands antilles or nicaragua or niger or nigeria or oman or muscat or pakistan or panama or papua new guinea or new guinea or paraguay or peru or philippines or philipines or phillipines or phillippines or poland or "polish people's republic" or portugal or portuguese republic or puerto rico or romania or russia or russian federation or ussr or soviet union or union of soviet socialist republics or rwanda or ruanda or samoa or pacific islands or polynesia or samoan islands or navigator island or navigator islands or "sao tome and principe" or saudi arabia or senegal or serbia or seychelles or sierra leone or slovakia or slovak republic or slovenia or melanesia or solomon island or solomon islands or norfolk island or norfolk islands or somalia or south africa or south sudan or sri lanka or ceylon or "saint kitts and

nevis" or "st. kitts and nevis" or saint lucia or "st. lucia" or "saint vincent and the grenadines" or saint vincent or "st. vincent" or grenadines or sudan or suriname or surinam or dutch guiana or netherlands guiana or syria or syrian arab republic or tajikistan or tadjikistan or tadzhikistan or tadzhik or tanzania or tanganyika or thailand or siam or timor leste or east timor or togo or togolese republic or tonga or "trinidad and tobago" or trinidad or tobago or tunisia or turkey or turkmenistan or turkmen or uganda or ukraine or uruguay or uzbekistan or uzbek or vanuatu or new hebrides or venezuela or vietnam or viet nam or middle east or west bank or gaza or palestine or yemen or yugoslavia or zambia or zimbabwe or northern rhodesia or global south or africa south of the sahara or sub-saharan africa or subsaharan africa or africa, central or central africa or africa, northern or north africa or northern africa or magreb or maghrib or sahara or africa, southern or southern africa or africa, eastern or east africa or eastern africa or africa, western or west africa or western africa or west indies or indian ocean islands or caribbean or central america or latin america or "south and central america" or south america or asia, central or central asia or asia, northern or north asia or northern asia or asia, southeastern or southeastern asia or south eastern asia or southeast asia or south east asia or asia, western or western asia or europe, eastern or east europe or eastern europe or developing country or developing countries or developing nation? or developing population? or developing world or less developed countr* or less developed nation? or less developed population? or less developed world or lesser developed countr* or lesser developed nation? or lesser developed population? or lesser developed world or under developed countr* or under developed nation? or under developed population? or under developed world or underdeveloped countr* or underdeveloped nation? or underdeveloped population? or underdeveloped world or middle income countr* or middle income nation? or middle income population? or low income countr* or low income nation? or low income population? or lower income countr* or lower income nation? or lower income population? or underserved countr* or underserved nation? or underserved population? or underserved world or under served countr* or under served nation? or under served population? or under served world or deprived countr* or deprived nation? or deprived population? or deprived world or poor countr* or poor nation? or poor population? or poor world or poorer countr* or poorer nation? or poorer population? or poorer world or developing econom* or less developed econom* or lesser developed econom* or under developed econom* or underdeveloped econom* or middle income econom* or low income econom* or lower income econom* or low gdp or low gnp or low gross domestic or low gross national or lower gdp or lower gnp or lower gross domestic or lower gross national or lmic or lmics or third world or lami countr* or transitional countr* or emerging economies or emerging nation?))
Expanders - Apply equivalent subjects
Search modes - Boolean/Phrase
Interface - EBSCOhost Research Databases
Search Screen - Advanced Search
Database - CINAHL 401,756

S2 AB (afghanistan or albania or algeria or american samoa or angola or "antigua and barbuda" or antigua or barbuda or argentina or armenia or armenian or aruba or azerbaijan or bahrain or bangladesh or barbados or republic of belarus or belarus or byelarus or belorussia or byelorussian or belize or british honduras or benin or dahomey or bhutan or bolivia or "bosnia and herzegovina" or bosnia or herzegovina or botswana or bechuanaland or brazil or brasil or bulgaria or burkina faso or burkina fasso or upper volta or burundi or urundi or cabo verde or cape verde or cambodia or kampuchea or khmer republic or cameroon or cameron or cameroun or central african republic or ubangi shari or chad or chile or china or colombia or comoros or comoro islands or iles comores or mayotte or democratic republic of the congo or democratic republic congo or congo or zaire or costa rica or "cote d'ivoire" or "cote d' ivoire" or cote divoire or cote d ivoire or ivory coast or croatia or cuba or cyprus or czech republic or czechoslovakia or djibouti or french somaliland or dominica or dominican republic or ecuador or egypt or united arab republic or el salvador or equatorial guinea or spanish guinea or eritrea or estonia or eswatini or swaziland or ethiopia or fiji or gabon or gabonese republic or gambia or "georgia (republic)" or georgian or ghana or gold coast or gibraltar or greece or grenada or guam or guatemala or guinea or guinea bissau or guyana or british guiana or haiti or hispaniola or honduras or hungary or india or indonesia or timor or iran or iraq or isle of man or jamaica or jordan or kazakhstan or kazakh or kenya or "democratic people's republic of korea" or republic of korea or north korea or south korea or korea or kosovo or kyrgyzstan or kirghizia or kirgizstan or kyrgyz

republic or kirghiz or laos or lao pdr or "lao people's democratic republic" or latvia or lebanon or lebanese republic or lesotho or basutoland or liberia or libya or libyan arab jamahiriya or lithuania or macau or macao or republic of north macedonia or macedonia or madagascar or malagasy republic or malawi or nyasaland or malaysia or malay federation or malaya federation or maldives or indian ocean islands or indian ocean or mali or malta or micronesia or federated states of micronesia or kiribati or marshall islands or nauru or northern mariana islands or palau or tuvalu or mauritania or mauritius or mexico or moldova or moldovian or mongolia or montenegro or morocco or ifni or mozambique or portuguese east africa or myanmar or burma or namibia or nepal or netherlands antilles or nicaragua or niger or nigeria or oman or muscat or pakistan or panama or papua new guinea or new guinea or paraguay or peru or philippines or philipines or phillipines or phillippines or poland or "polish people's republic" or portugal or portuguese republic or puerto rico or romania or russia or russian federation or ussr or soviet union or union of soviet socialist republics or rwanda or ruanda or samoa or pacific islands or polynesia or samoan islands or navigator island or navigator islands or "sao tome and principe" or saudi arabia or senegal or serbia or seychelles or sierra leone or slovakia or slovak republic or slovenia or melanesia or solomon island or solomon islands or norfolk island or norfolk islands or somalia or south africa or south sudan or sri lanka or ceylon or "saint kitts and nevis" or "st. kitts and nevis" or saint lucia or "st. lucia" or "saint vincent and the grenadines" or saint vincent or "st. vincent" or grenadines or sudan or suriname or surinam or dutch guiana or netherlands guiana or syria or syrian arab republic or tajikistan or tadjikistan or tadzhikistan or tadzhik or tanzania or tanganyika or thailand or siam or timor leste or east timor or togo or togolese republic or tonga or "trinidad and tobago" or trinidad or tobago or tunisia or turkey or turkmenistan or turkmen or uganda or ukraine or uruguay or uzbekistan or uzbek or vanuatu or new hebrides or venezuela or vietnam or viet nam or middle east or west bank or gaza or palestine or yemen or yugoslavia or zambia or zimbabwe or northern rhodesia or global south or africa south of the sahara or sub-saharan africa or subsaharan africa or africa, central or central africa or africa, northern or north africa or northern africa or magreb or maghrib or sahara or africa, southern or southern africa or africa, eastern or east africa or eastern africa or africa, western or west africa or western africa or west indies or indian ocean islands or caribbean or central america or latin america or "south and central america" or south america or asia, central or central asia or asia, northern or north asia or northern asia or asia, southeastern or southeastern asia or south eastern asia or southeast asia or south east asia or asia, western or western asia or europe, eastern or east europe or eastern europe or developing country or developing countries or developing nation? or developing population? or developing world or less developed countr* or less developed nation? or less developed population? or less developed world or lesser developed countr* or lesser developed nation? or lesser developed population? or lesser developed world or under developed countr* or under developed nation? or under developed population? or under developed world or underdeveloped countr* or underdeveloped nation? or underdeveloped population? or underdeveloped world or middle income countr* or middle income nation? or middle income population? or low income countr* or low income nation? or low income population? or lower income countr* or lower income nation? or lower income population? or underserved countr* or underserved nation? or underserved population? or underserved world or under served countr* or under served nation? or under served population? or under served world or deprived countr* or deprived nation? or deprived population? or deprived world or poor countr* or poor nation? or poor population? or poor world or poorer countr* or poorer nation? or poorer population? or poorer world or developing econom* or less developed econom* or lesser developed econom* or under developed econom* or underdeveloped econom* or middle income econom* or low income econom* or lower income econom* or low gdp or low gnp or low gross domestic or low gross national or lower gdp or lower gnp or lower gross domestic or lower gross national or lmic or lmics or third world or lami countr* or transitional countr* or emerging economies or emerging nation?)) OR AB ((afghanistan or albania or algeria or american samoa or angola or "antigua and barbuda" or antigua or barbuda or argentina or armenia or armenian or aruba or azerbaijan or bahrain or bangladesh or barbados or republic of belarus or belarus or byelarus or belorussia or byelorussian or belize or british honduras or benin or dahomey or bhutan or bolivia or "bosnia and herzegovina" or bosnia or herzegovina or botswana or bechuanaland or brazil or brasil or bulgaria or burkina faso or burkina fasso or upper volta or burundi or urundi or cabo verde or cape verde or cambodia or kampuchea or khmer republic or cameroon or cameron or cameroun or central african republic or ubangi shari or chad or chile or china or colombia or comoros or comoro islands or iles comores or mayotte or democratic republic of the congo or

democratic republic congo or congo or zaire or costa rica or "cote d’ivoire" or "cote d’ ivoire" or cote divoire or cote d ivoire or ivory coast or croatia or cuba or cyprus or czech republic or czechoslovakia or djibouti or french somaliland or dominica or dominican republic or ecuador or egypt or united arab republic or el salvador or equatorial guinea or spanish guinea or eritrea or estonia or eswatini or swaziland or ethiopia or fiji or gabon or gabonese republic or gambia or "georgia (republic)" or georgian or ghana or gold coast or gibraltar or greece or grenada or guam or guatemala or guinea or guinea bissau or guyana or british guiana or haiti or hispaniola or honduras or hungary or india or indonesia or timor or iran or iraq or isle of man or jamaica or jordan or kazakhstan or kazakh or kenya or "democratic people’s republic of korea" or republic of korea or north korea or south korea or korea or kosovo or kyrgyzstan or kirghizia or kirgizstan or kyrgyz republic or kirghiz or laos or lao pdr or "lao people's democratic republic" or latvia or lebanon or lebanese republic or lesotho or basutoland or liberia or libya or libyan arab jamahiriya or lithuania or macau or macao or republic of north macedonia or macedonia or madagascar or malagasy republic or malawi or nyasaland or malaysia or malay federation or malaya federation or maldives or indian ocean islands or indian ocean or mali or malta or micronesia or federated states of micronesia or kiribati or marshall islands or nauru or northern mariana islands or palau or tuvalu or mauritania or mauritius or mexico or moldova or moldovian or mongolia or montenegro or morocco or ifni or mozambique or portuguese east africa or myanmar or burma or namibia or nepal or netherlands antilles or nicaragua or niger or nigeria or oman or muscat or pakistan or panama or papua new guinea or new guinea or paraguay or peru or philippines or philipines or phillipines or phillippines or poland or "polish people's republic" or portugal or portuguese republic or puerto rico or romania or russia or russian federation or ussr or soviet union or union of soviet socialist republics or rwanda or ruanda or samoa or pacific islands or polynesia or samoan islands or navigator island or navigator islands or "sao tome and principe" or saudi arabia or senegal or serbia or seychelles or sierra leone or slovakia or slovak republic or slovenia or melanesia or solomon island or solomon islands or norfolk island or norfolk islands or somalia or south africa or south sudan or sri lanka or ceylon or "saint kitts and nevis" or "st. kitts and nevis" or saint lucia or "st. lucia" or "saint vincent and the grenadines" or saint vincent or "st. vincent" or grenadines or sudan or suriname or surinam or dutch guiana or netherlands guiana or syria or syrian arab republic or tajikistan or tadjikistan or tadzhikistan or tadzhik or tanzania or tanganyika or thailand or siam or timor leste or east timor or togo or togolese republic or tonga or "trinidad and tobago" or trinidad or tobago or tunisia or turkey or turkmenistan or turkmen or uganda or ukraine or uruguay or uzbekistan or uzbek or vanuatu or new hebrides or venezuela or vietnam or viet nam or middle east or west bank or gaza or palestine or yemen or yugoslavia or zambia or zimbabwe or northern rhodesia or global south or africa south of the sahara or sub-saharan africa or subsaharan africa or africa, central or central africa or africa, northern or north africa or northern africa or magreb or maghrib or sahara or africa, southern or southern africa or africa, eastern or east africa or eastern africa or africa, western or west africa or western africa or west indies or indian ocean islands or caribbean or central america or latin america or "south and central america" or south america or asia, central or central asia or asia, northern or north asia or northern asia or asia, southeastern or southeastern asia or south eastern asia or southeast asia or south east asia or asia, western or western asia or europe, eastern or east europe or eastern europe or developing country or developing countries or developing nation? or developing population? or developing world or less developed countr* or less developed nation? or less developed population? or less developed world or lesser developed countr* or lesser developed nation? or lesser developed population? or lesser developed world or under developed countr* or under developed nation? or under developed population? or under developed world or underdeveloped countr* or underdeveloped nation? or underdeveloped population? or underdeveloped world or middle income countr* or middle income nation? or middle income population? or low income countr* or low income nation? or low income population? or lower income countr* or lower income nation? or lower income population? or underserved countr* or underserved nation? or underserved population? or underserved world or under served countr* or under served nation? or under served population? or under served world or deprived countr* or deprived nation? or deprived population? or deprived world or poor countr* or poor nation? or poor population? or poor world or poorer countr* or poorer nation? or poorer population? or poorer world or developing econom* or less developed econom* or lesser developed econom* or under developed econom* or underdeveloped econom* or middle income econom* or low income econom* or lower income econom* or low gdp or low gnp or low gross domestic

or low gross national or lower gdp or lower gnp or lower gross domestic or lower gross national or lmic or lmics or third world or lami countr* or transitional countr* or emerging economies or emerging nation?))
Expanders - Apply equivalent subjects
Search modes - Boolean/Phrase
Interface - EBSCOhost Research Databases
Search Screen - Advanced Search
Database - CINAHL 303,391

S3 S1 OR S2
Expanders - Apply equivalent subjects
Search modes - Boolean/Phrase
Interface - EBSCOhost Research Databases
Search Screen - Advanced Search
Database - CINAHL 401,756

S4 TX ((youth* or young* or highschool* or high school* or student* or adolescent* or peer* or member* or kid* or teen* or coming of age) N3 (leader* or design* or facilitat* or develop* or initiat* or coordinat* or serve* or intervention* or initiative* or project* or program* or campaign* or promotion*))
Expanders - Apply equivalent subjects
Search modes - Boolean/Phrase
Interface - EBSCOhost Research Databases
Search Screen - Advanced Search
Database - CINAHL 115,819

S5 MH (MH "Peer Group") OR (MH "Group Exercise") OR (MH "Support Groups") OR (MH "Control Group") OR (MH "Nonequivalent Control Group") OR (MH "Known Groups") OR "peer group AND peer groups in adolescence"
Expanders - Apply equivalent subjects
Search modes - Boolean/Phrase
Interface - EBSCOhost Research Databases
Search Screen - Advanced Search
Database - CINAHL 42,115

S6 S4 OR S5
Expanders - Apply equivalent subjects
Search modes - Boolean/Phrase
Interface - EBSCOhost Research Databases
Search Screen - Advanced Search
Database - CINAHL 152,378

S7 MH (MH "Maternal-Child Health") OR (MH "Maternal Health Services") OR (MH "Postnatal Care") OR (MH "Prenatal Care") OR (MH "Prepregnancy Care") OR "Maternal Health"
Expanders - Apply equivalent subjects
Search modes - Boolean/Phrase
Interface - EBSCOhost Research Databases
Search Screen - Advanced Search
Database - CINAHL 39,398

S8 MH (MH "Pregnancy") OR "pregnancy" OR (MH "Pregnancy in Adolescence") OR (MH "Maternal Age 14 and Under") OR (MH "Pregnancy, Unplanned") OR (MH "Pregnancy Outcomes") OR (MH "Pregnancy Discomforts") OR (MH "Attitude to Pregnancy")
Expanders - Apply equivalent subjects
Search modes - Boolean/Phrase

Interface - EBSCOhost Research Databases
Search Screen - Advanced Search
Database - CINAHL 229,244

S9 "post-partum" OR (MH "Postpartum Hemorrhage") OR (MH "Stress Disorders, Post-Traumatic") OR (MH "Postnatal Period") OR (MH "Depression, Postpartum") OR (MH "Postpartum Care (Saba CCC)") OR (MH "Postpartum (Omaha)")
Expanders - Apply equivalent subjects
Search modes - Boolean/Phrase
Interface - EBSCOhost Research Databases
Search Screen - Advanced Search
Database - CINAHL 49,639

S10 ""peripartum""
Expanders - Apply equivalent subjects
Search modes - Boolean/Phrase
Interface - EBSCOhost Research Databases
Search Screen - Advanced Search
Database - CINAHL 2,356

S11 (MH "Pregnancy") OR "pregnan*" Expanders - Apply equivalent subjects
Search modes - Boolean/Phrase Interface - EBSCOhost Research Databases
Search Screen - Advanced Search
Database - CINAHL 280,411

S12 ""postpartum""
Expanders - Apply equivalent subjects
Search modes - Boolean/Phrase
Interface - EBSCOhost Research Databases
Search Screen - Advanced Search
Database - CINAHL 34,492

S13 ""maternal health""
Expanders - Apply equivalent subjects
Search modes - Boolean/Phrase
Interface - EBSCOhost Research Databases
Search Screen - Advanced Search
Database - CINAHL 14,904

S14 (MH "Fetal Well-Being") OR (MH "Psychological Well-Being") OR (MH "Well-Being (Iowa NOC)") OR "maternal wellbeing"
Expanders - Apply equivalent subjects
Search modes - Boolean/Phrase
Interface - EBSCOhost Research Databases
Search Screen - Advanced Search
Database - CINAHL 34,694

S15 neonat*
Expanders - Apply equivalent subjects
Search modes - Boolean/Phrase
Interface - EBSCOhost Research Databases
Search Screen - Advanced Search
Database - CINAHL 94,566

S16 S4 or S5 or S6 or S7 or S8 or S9 or S10 or S11 or S12 or S13 or S14 or S15
Expanders - Apply equivalent subjects
Search modes - Boolean/Phrase
Interface - EBSCOhost Research Databases
Search Screen - Advanced Search
Database - CINAHL 574,190

S17 S3 AND S6 AND S16
Search modes - Boolean/Phrase
Interface - EBSCOhost Research Databases
Search Screen - Advanced Search
Database - CINAHL 12,183

S18 S3 AND S6 AND S16
Limiters - Published Date: 20220101-20231231
Search modes - Boolean/Phrase
Interface - EBSCOhost Research Databases
Search Screen - Advanced Search
Database - CINAHL 1,295

Latin American and Caribbean Health Sciences literature (LILACS)
Database: 1982 to present
Search date: Jan 27, 2022
Used filter of LILACS; after many attempts, many combinations/iterations
(low-and-middle income countries develop* countr*) AND (youth intervention) AND (matern* neonat* health)
# of hits screened: 16

LILACS (update in 2023)
Database: 1982 to present
Search date: Jan 26, 2023
Used filter of LILACS; after many attempts, many combinations/iterations
(low-and-middle income countries develop* countr*) AND (youth intervention) AND (matern* neonat* health)
# of hits retrieved: 20
>added Publication Year Range: 2022 to 2023 as a filter, for final titles retrieved as 3 hits
# of hits screened: 3

Global Health
Database: Global Health <1910 to 2022 Week 07>
Search date: Feb 17, 2022
----
1 (afghanistan or albania or algeria or american samoa or angola or "antigua and barbuda" or antigua or barbuda or argentina or armenia or armenian or aruba or azerbaijan or bahrain or bangladesh or barbados or republic of belarus or belarus or byelarus or belorussia or byelorussian or belize or british honduras or benin or dahomey or bhutan or bolivia or "bosnia and herzegovina" or bosnia or herzegovina or botswana or bechuanaland or brazil or brasil or bulgaria or burkina faso or burkina fasso or upper volta or burundi or urundi or cabo verde or cape verde or cambodia or kampuchea or khmer republic or cameroon or cameron or cameroun or central african republic or ubangi shari or chad or chile or china or colombia or comoros or comoro islands or iles comores or mayotte or democratic republic of the congo or democratic republic congo or congo or zaire or costa rica or "cote d’ivoire" or "cote d’ ivoire" or cote divoire or cote d ivoire or ivory coast or croatia or cuba or cyprus or czech republic or czechoslovakia or djibouti or french somaliland

or dominica or dominican republic or ecuador or egypt or united arab republic or el salvador or equatorial guinea or spanish guinea or eritrea or estonia or eswatini or swaziland or ethiopia or fiji or gabon or gabonese republic or gambia or "georgia (republic)" or georgian or ghana or gold coast or gibraltar or greece or grenada or guam or guatemala or guinea or guinea bissau or guyana or british guiana or haiti or hispaniola or honduras or hungary or india or indonesia or timor or iran or iraq or isle of man or jamaica or jordan or kazakhstan or kazakh or kenya or "democratic people's republic of korea" or republic of korea or north korea or south korea or korea or kosovo or kyrgyzstan or kirghizia or kirgizstan or kyrgyz republic or kirghiz or laos or lao pdr or "lao people's democratic republic" or latvia or lebanon or lebanese republic or lesotho or basutoland or liberia or libya or libyan arab jamahiriya or lithuania or macau or macao or republic of north macedonia or macedonia or madagascar or malagasy republic or malawi or nyasaland or malaysia or malay federation or malaya federation or maldives or indian ocean islands or indian ocean or mali or malta or micronesia or federated states of micronesia or kiribati or marshall islands or nauru or northern mariana islands or palau or tuvalu or mauritania or mauritius or mexico or moldova or moldovian or mongolia or montenegro or "montenegro (republic)" or morocco or ifni or mozambique or portuguese east africa or myanmar or burma or namibia or nepal or netherlands antilles or nicaragua or niger or nigeria or oman or muscat or pakistan or panama or papua new guinea or new guinea or paraguay or peru or philippines or philipines or phillipines or phillippines or poland or "polish people's republic" or portugal or portuguese republic or puerto rico or romania or russia or russian federation or ussr or soviet union or union of soviet socialist republics or rwanda or ruanda or samoa or pacific islands or polynesia or samoan islands or navigator island or navigator islands or "sao tome and principe" or saudi arabia or senegal or serbia or seychelles or sierra leone or slovakia or slovak republic or slovenia or melanesia or solomon island or solomon islands or norfolk island or norfolk islands or somalia or south africa or south sudan or sri lanka or ceylon or "saint kitts and nevis" or "st. kitts and nevis" or saint lucia or "st. lucia" or "saint vincent and the grenadines" or saint vincent or "st. vincent" or grenadines or sudan or suriname or surinam or dutch guiana or netherlands guiana or syria or syrian arab republic or tajikistan or tadjikistan or tadzhikistan or tadzhik or tanzania or tanganyika or thailand or siam or timor leste or east timor or togo or togolese republic or tonga or "trinidad and tobago" or trinidad or tobago or tunisia or "turkey (republic)" or turkey or turkmenistan or turkmen or uganda or ukraine or uruguay or uzbekistan or uzbek or vanuatu or new hebrides or venezuela or vietnam or viet nam or middle east or west bank or gaza or palestine or yemen or yugoslavia or zambia or zimbabwe or northern rhodesia or global south or africa south of the sahara or "sub saharan africa" or subsaharan africa or africa, central or central africa or africa, northern or north africa or northern africa or magreb or maghrib or sahara or africa, southern or southern africa or africa, eastern or east africa or eastern africa or africa, western or west africa or western africa or west indies or indian ocean islands or caribbean region or caribbean islands or caribbean or central america or latin america or "south and central america" or south america or asia, central or central asia or asia, northern or north asia or northern asia or asia, southeastern or southeastern asia or south eastern asia or southeast asia or south east asia or asia, western or western asia or europe, eastern or east europe or eastern europe or developing country or developing countries or developing nation? or developing population? or developing world or less developed countr* or less developed nation? or less developed population? or less developed world or lesser developed countr* or lesser developed nation? or lesser developed population? or lesser developed world or under developed countr* or under developed nation? or under developed population? or under developed world or underdeveloped countr* or underdeveloped nation? or underdeveloped population? or underdeveloped world or middle income countr* or middle income nation? or middle income population? or low income countr* or low income nation? or low income population? or lower income countr* or lower income nation? or lower income population? or underserved countr* or underserved nation? or underserved population? or underserved world or under served countr* or under served nation? or under served population? or under served world or deprived countr* or deprived nation? or deprived population? or deprived world or poor countr* or poor nation? or poor population? or poor world or poorer countr* or poorer nation? or poorer population? or poorer world or developing econom* or less developed econom* or lesser developed econom* or under developed econom* or underdeveloped econom* or middle income econom* or low income econom* or lower income econom* or low gdp or low gnp or low gross domestic or low gross national or lower gdp or lower gnp or lower gross domestic or lower gross national or lmic or lmics or third

world or lami countr* or transitional countr* or emerging economies or emerging nation?).ti,ab,sh. (1415550)
Annotation: Had to take off: kw. and/or kf. for Global Health database.
2 ((youth* or young* or highschool* or high school* or student* or adolescent* or peer* or member* or kid* or teen* or coming of age) adj3 (leader* or design* or facilitat* or develop* or initiat* or coordinat* or serve* or intervention* or initiative* or project* or program* or campaign* or promotion*)).ti,ab. (25247)
3 1 and 2 (8480)
4 pregnan*.mp. (149095)
Annotation: Should I be using other symbols for truncation (limited and unlimited) and wildcards?
5 postpartum.mp. (13885)
6 Peripartum.mp. (774)
7 Maternal Health.mp. (4600)
8 maternal wellbeing.mp. (28)
9 maternal well-being.mp. (56)
10 neonat*.mp. (68285)
11 4 or 5 or 6 or 7 or 8 or 9 or 10 (201610)
12 3 and 11 (769)

Global Health (update in 2023)
Database: Global Health <1910 to 2023 Week 03>
Search date: Jan 23, 2023
----
1 (afghanistan or albania or algeria or american samoa or angola or "antigua and barbuda" or antigua or barbuda or argentina or armenia or armenian or aruba or azerbaijan or bahrain or bangladesh or barbados or republic of belarus or belarus or byelarus or belorussia or byelorussian or belize or british honduras or benin or dahomey or bhutan or bolivia or "bosnia and herzegovina" or bosnia or herzegovina or botswana or bechuanaland or brazil or brasil or bulgaria or burkina faso or burkina fasso or upper volta or burundi or urundi or cabo verde or cape verde or cambodia or kampuchea or khmer republic or cameroon or cameron or cameroun or central african republic or ubangi shari or chad or chile or china or colombia or comoros or comoro islands or iles comores or mayotte or democratic republic of the congo or democratic republic congo or congo or zaire or costa rica or "cote d’ivoire" or "cote d’ ivoire" or cote divoire or cote d ivoire or ivory coast or croatia or cuba or cyprus or czech republic or czechoslovakia or djibouti or french somaliland or dominica or dominican republic or ecuador or egypt or united arab republic or el salvador or equatorial guinea or spanish guinea or eritrea or estonia or eswatini or swaziland or ethiopia or fiji or gabon or gabonese republic or gambia or "georgia (republic)" or georgian or ghana or gold coast or gibraltar or greece or grenada or guam or guatemala or guinea or guinea bissau or guyana or british guiana or haiti or hispaniola or honduras or hungary or india or indonesia or timor or iran or iraq or isle of man or jamaica or jordan or kazakhstan or kazakh or kenya or "democratic people’s republic of korea" or republic of korea or north korea or south korea or korea or kosovo or kyrgyzstan or kirghizia or kirgizstan or kyrgyz republic or kirghiz or laos or lao pdr or "lao people's democratic republic" or latvia or lebanon or lebanese republic or lesotho or basutoland or liberia or libya or libyan arab jamahiriya or lithuania or macau or macao or republic of north macedonia or macedonia or madagascar or malagasy republic or malawi or nyasaland or malaysia or malay federation or malaya federation or maldives or indian ocean islands or indian ocean or mali or malta or micronesia or federated states of micronesia or kiribati or marshall islands or nauru or northern mariana islands or palau or tuvalu or mauritania or mauritius or mexico or moldova or moldovian or mongolia or montenegro or "montenegro (republic)" or morocco or ifni or mozambique or portuguese east africa or myanmar or burma or namibia or nepal or netherlands antilles or nicaragua or niger or nigeria or oman or muscat or pakistan or panama or papua new guinea or new guinea or paraguay or peru or philippines or philipines or phillipines or phillippines or poland or "polish people's republic" or portugal or portuguese republic or puerto rico or romania or russia or russian federation or ussr or soviet union or union of soviet socialist republics or rwanda or ruanda or samoa or pacific islands or polynesia or samoan islands or navigator island or navigator islands or "sao tome and principe" or saudi arabia or senegal or serbia or seychelles or sierra leone or slovakia or slovak republic or slovenia or melanesia or solomon island or

solomon islands or norfolk island or norfolk islands or somalia or south africa or south sudan or sri lanka or ceylon or "saint kitts and nevis" or "st. kitts and nevis" or saint lucia or "st. lucia" or "saint vincent and the grenadines" or saint vincent or "st. vincent" or grenadines or sudan or suriname or surinam or dutch guiana or netherlands guiana or syria or syrian arab republic or tajikistan or tadjikistan or tadzhikistan or tadzhik or tanzania or tanganyika or thailand or siam or timor leste or east timor or togo or togolese republic or tonga or "trinidad and tobago" or trinidad or tobago or tunisia or "turkey (republic)" or turkey or turkmenistan or turkmen or uganda or ukraine or uruguay or uzbekistan or uzbek or vanuatu or new hebrides or venezuela or vietnam or viet nam or middle east or west bank or gaza or palestine or yemen or yugoslavia or zambia or zimbabwe or northern rhodesia or global south or africa south of the sahara or "sub saharan africa" or subsaharan africa or africa, central or central africa or africa, northern or north africa or northern africa or magreb or maghrib or sahara or africa, southern or southern africa or africa, eastern or east africa or eastern africa or africa, western or west africa or western africa or west indies or indian ocean islands or caribbean region or caribbean islands or caribbean or central america or latin america or "south and central america" or south america or asia, central or central asia or asia, northern or north asia or northern asia or asia, southeastern or southeastern asia or south eastern asia or southeast asia or south east asia or asia, western or western asia or europe, eastern or east europe or eastern europe or developing country or developing countries or developing nation? or developing population? or developing world or less developed countr* or less developed nation? or less developed population? or less developed world or lesser developed countr* or lesser developed nation? or lesser developed population? or lesser developed world or under developed countr* or under developed nation? or under developed population? or under developed world or underdeveloped countr* or underdeveloped nation? or underdeveloped population? or underdeveloped world or middle income countr* or middle income nation? or middle income population? or low income countr* or low income nation? or low income population? or lower income countr* or lower income nation? or lower income population? or underserved countr* or underserved nation? or underserved population? or underserved world or under served countr* or under served nation? or under served population? or under served world or deprived countr* or deprived nation? or deprived population? or deprived world or poor countr* or poor nation? or poor population? or poor world or poorer countr* or poorer nation? or poorer population? or poorer world or developing econom* or less developed econom* or lesser developed econom* or under developed econom* or underdeveloped econom* or middle income econom* or low income econom* or lower income econom* or low gdp or low gnp or low gross domestic or low gross national or lower gdp or lower gnp or lower gross domestic or lower gross national or lmic or lmics or third world or lami countr* or transitional countr* or emerging economies or emerging nation?).ti,ab,sh. (1492445)
Annotation: Had to take off: kw. and/or kf. for Global Health database.
2 ((youth* or young* or highschool* or high school* or student* or adolescent* or peer* or member* or kid* or teen* or coming of age) adj3 (leader* or design* or facilitat* or develop* or initiat* or coordinat* or serve* or intervention* or initiative* or project* or program* or campaign* or promotion*)).ti,ab. (27321)
3 1 and 2 (9358)
4 pregnan*.mp. (158259)
Annotation: Should I be using other symbols for truncation (limited and unlimited) and wildcards?
5 postpartum.mp. (15129)
6 Peripartum.mp. (855)
7 Maternal Health.mp. (5017)
8 maternal wellbeing.mp. (30)
9 maternal well-being.mp. (63)
10 neonat*.mp. (72059)
11 4 or 5 or 6 or 7 or 8 or 9 or 10 (213251)
12 3 and 11 (847)
13 limit 12 to yr="2022 - 2023" (59)

Web of Science
Database: WOS 1976 to present
Search date: Jan 24, 2022

TS=(afghanistan or albania or algeria or american samoa or angola or "antigua and barbuda" or antigua or barbuda or argentina or armenia or armenia aruba or azerbaijan or bahrain or bangladesh or barbados or republic of belarus or belarus or byelarus or belorussia or byelorussian or belize or britis honduras or benin or dahomey or bhutan or bolivia or "bosnia and herzegovina" or bosnia or herzegovina or botswana or bechuanaland or brazil or brasil or bulgaria or burkina faso or burkina fasso or upper volta or burundi or urundi or cabo verde or cape verde or cambodia or kampuchea or khm republic or cameroon or cameron or cameroun or central african republic or ubangi shari or chad or chile or china or colombia or comoros or comoro islands or iles comores or mayotte or democratic republic of the congo or democratic republic congo or congo or zaire or costa rica or "cote divoire" o "cote d ivoire" or cote divoire or cote d ivoire or ivory coast or croatia or cuba or cyprus or czech republic or czechoslovakia or djibouti or french somaliland or dominica or dominican republic or ecuador or egypt or united arab republic or el salvador or equatorial guinea or spanish guinea or eritrea or estonia or eswatini or swaziland or ethiopia or fiji or gabon or gabonese republic or gambia or "georgia (republic) " or georgian or ghana or gold coast or gibraltar or greece or grenada or guam or guatemala or guinea or guinea bissau or guyana or british guiana or haiti or hispaniola or honduras or hungary or india or indonesia or timor or iran or iraq or isle of man or jamaica or jordan or kazakhstan or kazakh or kenya or "democratic peoples republic of korea" or republic of korea or north korea or south korea or korea or kosovo or kyrgyzstan or kirghizia or kirgizstan or kyrgyz republic or kirghiz or laos or lao pdr or "lao people's democratic republic" or latvia or lebanon or lebanese republic or lesotho or basutoland or liberia or libya or libyan arab jamahiriya or lithuania or macau or macao or republic of north macedonia or macedonia or madagascar malagasy republic or malawi or nyasaland or malaysia or malay federation or malaya federation or maldives or indian ocean islands or indian ocean mali or malta or micronesia or federated states of micronesia or kiribati or marshall islands or nauru or northern mariana islands or palau or tuvalu o mauritania or mauritius or mexico or moldova or moldovian or mongolia or montenegro or morocco or ifni or mozambique or portuguese east africa myanmar or burma or namibia or nepal or netherlands antilles or nicaragua or niger or nigeria or oman or muscat or pakistan or panama or papua ne guinea or new guinea or paraguay or peru or philippines or philipines or phillipines or phillippines or poland or "polish people's republic" or portuga or portuguese republic or puerto rico or romania or russia or russian federation or ussr or soviet union or union of soviet socialist republics or rwanda or ruanda or samoa or pacific islands or polynesia or samoan islands or navigator island or navigator islands or "sao tome and principe" or saudi ara or senegal or serbia or seychelles or sierra leone or slovakia or slovak republic or slovenia or melanesia or solomon island or solomon islands or norf island or norfolk islands or somalia or south africa or south sudan or sri lanka or ceylon or "saint kitts and nevis" or "st. kitts and nevis" or saint lucia "st. lucia" or "saint vincent and the grenadines" or saint vincent or "st. vincent" or grenadines or sudan or suriname or surinam or dutch guiana or netherlands guiana or syria or syrian arab republic or tajikistan or tadjikistan or tadzhikistan or tadzhik or tanzania or tanganyika or thailand or siam timor leste or east timor or togo or togolese republic or tonga or "trinidad and tobago" or trinidad or tobago or tunisia or turkey or turkmenistan or turkmen or uganda or ukraine or uruguay or uzbekistan or uzbek or vanuatu or new hebrides or venezuela or vietnam or viet nam or middle east or west bank or gaza or palestine or yemen or yugoslavia or zambia or zimbabwe or northern rhodesia or global south or africa south of the sahara or sub-saharan africa or subsaharan africa or africa, central or central africa or africa, northern or north africa or northern africa or magreb or maghrib o sahara or africa, southern or southern africa or africa, eastern or east africa or eastern africa or africa, western or west africa or western africa or west indies or indian ocean islands or caribbean or central america or latin america or "south and central america" or south america or asia, central or central asia or asia, northern or north asia or northern asia or asia, southeastern or southeastern asia or south eastern asia or southeast asia or south east asia or asia, western or western asia or europe, eastern or east europe or eastern europe or developing country or developing countries or developing nation? or developing population? or developing world or less developed countr* or less developed nation? or less developed population or less developed world or lesser developed countr* or lesser developed nation? or lesser developed population? or lesser developed world or under developed countr* or under developed nation? or under developed population? or under developed world or underdeveloped countr* or underdeveloped nation? or underdeveloped population? or underdeveloped world or middle income countr* or middle income nation? or middle income population? or low income

countr* or low income nation? or low income population? or lower income countr* or lower income nation? or lower income population? or underserved countr* or underserved nation? or underserved population? or underserved world or under served countr or under served nation? or under served population? or under served world or deprived countr* or deprived nation? or deprived population? or deprived world or poor countr* or poor nation? or poor population? or poor world or poorer countr* or poorer nation? or poorer population? or poor world or developing econom* or less developed econom* or lesser developed econom* or under developed econom* or underdeveloped econom* o middle income econom* or low income econom* or lower income econom* or low gdp or low gnp or low gross domestic or low gross national or low gdp or lower gnp or lower gross domestic or lower gross national or lmic or lmics or third world or lami countr* or transitional countr* or emerging economies or emerging nation?)

TS=((youth* or young* or highschool* or "high school*" or student* or adolescent* or peer* or member* or kid* or teen* or "coming of age") NEAR/3 (leader* or design* or facilitat* or develop* or initiat* or coordinat* or serve* or intervention* or initiative* or project* or program* or campaign* or promotion*))

TS=("Maternal Health" OR Pregnancy OR "postpartum period" OR "Peripartum Period" OR pregnan* OR postpartum OR Peripartum OR “Maternal Health" OR “maternal wellbeing” OR “maternal well-being” OR neonat* or newborn or infant or preterm* or prem* or bab*)

4
#1 AND #2 AND #3
2,751
Add to query

3
TS=(afghanistan or albania or algeria or american samoa or angola or "antigua and barbuda" or antigua or barbuda or argentina or armenia or armenia aruba or azerbaijan or bahrain or bangladesh or barbados or republic of belarus or belarus or byelarus or belorussia or byelorussian or belize or britis honduras or benin or dahomey or bhutan or bolivia or "bosnia and herzegovina" or bosnia or herzegovina or botswana or bechuanaland or brazil or brasil or bulgaria or burkina faso or burkina fasso or upper volta or burundi or urundi or cabo verde or cape verde or cambodia or kampuchea or khm republic or cameroon or cameron or cameroun or central african republic or ubangi shari or chad or chile or china or colombia or comoros or comoro islands or iles comores or mayotte or democratic republic of the congo or democratic republic congo or congo or zaire or costa rica or "cote divoire" o "cote d ivoire" or cote divoire or cote d ivoire or ivory coast or croatia or cuba or cyprus or czech republic or czechoslovakia or djibouti or french somaliland or dominica or dominican republic or ecuador or egypt or united arab republic or el salvador or equatorial guinea or spanish guinea or eritrea or estonia or eswatini or swaziland or ethiopia or fiji or gabon or gabonese republic or gambia or "georgia (republic) " or georgian or ghana or gold coast or gibraltar or greece or grenada or guam or guatemala or guinea or guinea bissau or guyana or british guiana or haiti or hispaniola or honduras or hungary or india or indonesia or timor or iran or iraq or isle of man or jamaica or jordan or kazakhstan or kazakh or kenya or "democratic peoples republic of korea" or republic of korea or north korea or south korea or korea or kosovo or kyrgyzstan or kirghizia or kirgizstan or kyrgyz republic or kirghiz or laos or lao pdr or "lao people's democratic republic" or latvia or lebanon or lebanese republic or lesotho or basutoland or liberia or libya or libyan arab jamahiriya or lithuania or macau or macao or republic of north macedonia or macedonia or madagascar malagasy republic or malawi or nyasaland or malaysia or malay federation or malaya federation or maldives or indian ocean islands or indian ocean mali or malta or micronesia or federated states of micronesia or kiribati or marshall islands or nauru or northern mariana islands or palau or tuvalu o mauritania or mauritius or mexico or moldova or moldovian or mongolia or montenegro or morocco or ifni or mozambique or portuguese east africa myanmar or burma or namibia or nepal or netherlands antilles or nicaragua or niger or nigeria or oman or muscat or pakistan or panama or papua ne guinea or new guinea or paraguay or peru or philippines or philipines or phillipines or phillippines or poland or "polish people's republic" or portuga or portuguese republic or puerto rico or romania or russia or russian federation or ussr or soviet union or union of soviet socialist republics or rwanda or ruanda or

samoa or pacific islands or polynesia or samoan islands or navigator island or navigator islands or "sao tome and principe" or saudi ara or senegal or serbia or seychelles or sierra leone or slovakia or slovak republic or slovenia or melanesia or solomon island or solomon islands or norf island or norfolk islands or somalia or south africa or south sudan or sri lanka or ceylon or "saint kitts and nevis" or "st. kitts and nevis" or saint lucia "st. lucia" or "saint vincent and the grenadines" or saint vincent or "st. vincent" or grenadines or sudan or suriname or surinam or dutch guiana or netherlands guiana or syria or syrian arab republic or tajikistan or tadjikistan or tadzhikistan or tadzhik or tanzania or tanganyika or thailand or siam timor leste or east timor or togo or togolese republic or tonga or "trinidad and tobago" or trinidad or tobago or tunisia or turkey or turkmenistan or turkmen or uganda or ukraine or uruguay or uzbekistan or uzbek or vanuatu or new hebrides or venezuela or vietnam or viet nam or middle east or west bank or gaza or palestine or yemen or yugoslavia or zambia or zimbabwe or northern rhodesia or global south or africa south of the sahara or sub-saharan africa or subsaharan africa or africa, central or central africa or africa, northern or north africa or northern africa or magreb or maghrib o sahara or africa, southern or southern africa or africa, eastern or east africa or eastern africa or africa, western or west africa or western africa or west indies or indian ocean islands or caribbean or central america or latin america or "south and central america" or south america or asia, central or central asia or asia, northern or north asia or northern asia or asia, southeastern or southeastern asia or south eastern asia or southeast asia or south east asia or asia, western or western asia or europe, eastern or east europe or eastern europe or developing country or developing countries or developing nation? or developing population? or developing world or less developed countr* or less developed nation? or less developed population or less developed world or lesser developed countr* or lesser developed nation? or lesser developed population? or lesser developed world or under developed countr* or under developed nation? or under developed population? or under developed world or underdeveloped countr* or underdeveloped nation? or underdeveloped population? or underdeveloped world or middle income countr* or middle income nation? or middle income population? or low income countr* or low income nation? or low income population? or lower income countr* or lower income nation? or lower income population? or underserved countr* or underserved nation? or underserved population? or underserved world or under served countr or under served nation? or under served population? or under served world or deprived countr* or deprived nation? or deprived population? or deprived world or poor countr* or poor nation? or poor population? or poor world or poorer countr* or poorer nation? or poorer population? or poor world or developing econom* or less developed econom* or lesser developed econom* or under developed econom* or underdeveloped econom* o middle income econom* or low income econom* or lower income econom* or low gdp or low gnp or low gross domestic or low gross national or low gdp or lower gnp or lower gross domestic or lower gross national or lmic or lmics or third world or lami countr* or transitional countr* or emerging economies or emerging nation?)
4,886,513
Add to query

2
TS=((youth* or young* or highschool* or "high school*" or student* or adolescent* or peer* or member* or kid* or teen* or "coming of age") NEAR/3 (leader* or design* or facilitat* or develop* or initiat* or coordinat* or serve* or intervention* or initiative* or project* or program* or campaign* or promotion*))
270,998
Add to query

1
TS=("Maternal Health" OR Pregnancy OR "postpartum period" OR "Peripartum Period" OR pregnan* OR postpartum OR Peripartum OR “Maternal Health" OR “maternal wellbeing” OR “maternal well-being” OR neonat* or newborn or infant or preterm* or prem* or bab*)
1,645,283

Web of Science (update in 2023)
Database: Web of Science using limiter of 2022-2023 publication years
Search date: Jan 2, 2023

5
#1 AND #2 AND #3 and 2022 or 2023 (Publication Years)
262

4
#1 AND #2 AND #3
3,007

3
TS=(afghanistan or albania or algeria or american samoa or angola or "antigua and barbuda" or antigua or barbuda or argentina or armenia or armenia aruba or azerbaijan or bahrain or bangladesh or barbados or republic of belarus or belarus or byelarus or belorussia or byelorussian or belize or britis honduras or benin or dahomey or bhutan or bolivia or "bosnia and herzegovina" or bosnia or herzegovina or botswana or bechuanaland or brazil or brasil or bulgaria or burkina faso or burkina fasso or upper volta or burundi or urundi or cabo verde or cape verde or cambodia or kampuchea or khm republic or cameroon or cameron or cameroun or central african republic or ubangi shari or chad or chile or china or colombia or comoros or comoro islands or iles comores or mayotte or democratic republic of the congo or democratic republic congo or congo or zaire or costa rica or "cote divoire" o "cote d ivoire" or cote divoire or cote d ivoire or ivory coast or croatia or cuba or cyprus or czech republic or czechoslovakia or djibouti or french somaliland or dominica or dominican republic or ecuador or egypt or united arab republic or el salvador or equatorial guinea or spanish guinea or eritrea or estonia or eswatini or swaziland or ethiopia or fiji or gabon or gabonese republic or gambia or "georgia (republic) " or georgian or ghana or gold coast or gibraltar or greece or grenada or guam or guatemala or guinea or guinea bissau or guyana or british guiana or haiti or hispaniola or honduras or hungary or india or indonesia or timor or iran or iraq or isle of man or jamaica or jordan or kazakhstan or kazakh or kenya or "democratic peoples republic of korea" or republic of korea or north korea or south korea or korea or kosovo or kyrgyzstan or kirghizia or kirgizstan or kyrgyz republic or kirghiz or laos or lao pdr or "lao people's democratic republic" or latvia or lebanon or lebanese republic or lesotho or basutoland or liberia or libya or libyan arab jamahiriya or lithuania or macau or macao or republic of north macedonia or macedonia or madagascar malagasy republic or malawi or nyasaland or malaysia or malay federation or malaya federation or maldives or indian ocean islands or indian ocean mali or malta or micronesia or federated states of micronesia or kiribati or marshall islands or nauru or northern mariana islands or palau or tuvalu o mauritania or mauritius or mexico or moldova or moldovian or mongolia or montenegro or morocco or ifni or mozambique or portuguese east africa myanmar or burma or namibia or nepal or netherlands antilles or nicaragua or niger or nigeria or oman or muscat or pakistan or panama or papua ne guinea or new guinea or paraguay or peru or philippines or philipines or phillipines or phillippines or poland or "polish people's republic" or portuga or portuguese republic or puerto rico or romania or russia or russian federation or ussr or soviet union or union of soviet socialist republics or rwanda or ruanda or samoa or pacific islands or polynesia or samoan islands or navigator island or navigator islands or "sao tome and principe" or saudi ara or senegal or serbia or seychelles or sierra leone or slovakia or slovak republic or slovenia or melanesia or solomon island or solomon islands or norf island or norfolk islands or somalia or south africa or south sudan or sri lanka or ceylon or "saint kitts and nevis" or "st. kitts and nevis" or saint lucia "st. lucia" or "saint vincent and the grenadines" or saint vincent or "st. vincent" or grenadines or sudan or suriname or surinam or dutch guiana or netherlands guiana or syria or syrian arab republic or tajikistan or tadjikistan or tadzhikistan or tadzhik or tanzania or tanganyika or thailand or siam timor leste or east timor or togo or togolese republic or tonga or "trinidad and tobago" or trinidad or tobago or tunisia or turkey or turkmenistan or turkmen or uganda or ukraine or uruguay or uzbekistan or uzbek or vanuatu or new hebrides or venezuela or vietnam or viet nam or middle east or west bank or gaza or palestine or yemen or yugoslavia or zambia or zimbabwe or northern rhodesia or global south or africa south of the sahara or sub-saharan africa or subsaharan africa or africa, central or central africa or africa, northern or north africa or northern africa or magreb or maghrib o sahara or africa, southern or southern africa or africa, eastern or east africa or eastern africa or africa, western or west africa or western africa or west indies or indian ocean islands or caribbean or central america or latin america or "south and central america" or south america or asia, central or central asia or asia, northern or north asia or northern asia or asia, southeastern or

southeastern asia or south eastern asia or southeast asia or south east asia or asia, western or western asia or europe, eastern or east europe or eastern europe or developing country or developing countries or developing nation? or developing population? or developing world or less developed countr* or less developed nation? or less developed population or less developed world or lesser developed countr* or lesser developed nation? or lesser developed population? or lesser developed world or under developed countr* or under developed nation? or under developed population? or under developed world or underdeveloped countr* or underdeveloped nation? or underdeveloped population? or underdeveloped world or middle income countr* or middle income nation? or middle income population? or low income countr* or low income nation? or low income population? or lower income countr* or lower income nation? or lower income population? or underserved countr* or underserved nation? or underserved population? or underserved world or under served countr or under served nation? or under served population? or under served world or deprived countr* or deprived nation? or deprived population? or deprived world or poor countr* or poor nation? or poor population? or poor world or poorer countr* or poorer nation? or poorer population? or poor world or developing econom* or less developed econom* or lesser developed econom* or under developed econom* or underdeveloped econom* o middle income econom* or low income econom* or lower income econom* or low gdp or low gnp or low gross domestic or low gross national or low gdp or lower gnp or lower gross domestic or lower gross national or lmic or lmics or third world or lami countr* or transitional countr* or emerging economies or emerging nation?)
5,263,871

2
TS=((youth* or young* or highschool* or "high school*" or student* or adolescent* or peer* or member* or kid* or teen* or "coming of age") NEAR/3 (leader* or design* or facilitat* or develop* or initiat* or coordinat* or serve* or intervention* or initiative* or project* or program* or campaign* or promotion*))
293,772
1
TS=("Maternal Health" OR Pregnancy OR "postpartum period" OR "Peripartum Period" OR pregnan* OR postpartum OR Peripartum OR “Maternal Health" OR “maternal wellbeing” OR “maternal well-being” OR neonat* or newborn or infant or preterm* or prem* or bab*)
1,732,758

Cochrane Central Register of Controlled Trials (CENTRAL)
Database: CENTRAL to present
Search date: Jan 27, 2022
Applied Search limit/filter: >content type>trials

Concept 1 in Line 1: # hits: 289, 695
afghanistan or albania or algeria or american samoa or angola or "antigua and barbuda" or antigua or barbuda or argentina or armenia or armenia aruba or azerbaijan or bahrain or bangladesh or barbados or republic of belarus or belarus or byelarus or belorussia or byelorussian or belize or britis honduras or benin or dahomey or bhutan or bolivia or "bosnia and herzegovina" or bosnia or herzegovina or botswana or bechuanaland or brazil or brasil or bulgaria or burkina faso or burkina fasso or upper volta or burundi or urundi or cabo verde or cape verde or cambodia or kampuchea or khm republic or cameroon or cameron or cameroun or central african republic or ubangi shari or chad or chile or china or colombia or comoros or comoro islands or iles comores or mayotte or democratic republic of the congo or democratic republic congo or congo or zaire or costa rica or "cote divoire" o "cote d ivoire" or cote divoire or cote d ivoire or ivory coast or croatia or cuba or cyprus or czech republic or czechoslovakia or djibouti or french somaliland or dominica or dominican republic or ecuador or egypt or united arab republic or el salvador or equatorial guinea or spanish guinea or eritrea or estonia or eswatini or swaziland or ethiopia or fiji or gabon or gabonese republic or gambia or "georgia (republic) " or georgian or ghana or gold coast or gibraltar or greece or grenada or guam or guatemala or guinea or guinea bissau or guyana or british guiana or haiti or hispaniola or honduras or hungary or india or indonesia or timor or iran or iraq or isle of man or jamaica or jordan or kazakhstan or kazakh or kenya or "democratic peoples republic of korea" or republic of korea or

north korea or south korea or korea or kosovo or kyrgyzstan or kirghizia or kirgizstan or kyrgyz republic or kirghiz or laos or lao pdr or "lao people's democratic republic" or latvia or lebanon or lebanese republic or lesotho or basutoland or liberia or libya or libyan arab jamahiriya or lithuania or macau or macao or republic of north macedonia or macedonia or madagascar malagasy republic or malawi or nyasaland or malaysia or malay federation or malaya federation or maldives or indian ocean islands or indian ocean mali or malta or micronesia or federated states of micronesia or kiribati or marshall islands or nauru or northern mariana islands or palau or tuvalu o mauritania or mauritius or mexico or moldova or moldovian or mongolia or montenegro or morocco or ifni or mozambique or portuguese east africa myanmar or burma or namibia or nepal or netherlands antilles or nicaragua or niger or nigeria or oman or muscat or pakistan or panama or papua ne guinea or new guinea or paraguay or peru or philippines or philipines or phillipines or phillippines or poland or "polish people's republic" or portuga or portuguese republic or puerto rico or romania or russia or russian federation or ussr or soviet union or union of soviet socialist republics or rwanda or ruanda or samoa or pacific islands or polynesia or samoan islands or navigator island or navigator islands or "sao tome and principe" or saudi ara or senegal or serbia or seychelles or sierra leone or slovakia or slovak republic or slovenia or melanesia or solomon island or solomon islands or norf island or norfolk islands or somalia or south africa or south sudan or sri lanka or ceylon or "saint kitts and nevis" or "st. kitts and nevis" or saint lucia "st. lucia" or "saint vincent and the grenadines" or saint vincent or "st. vincent" or grenadines or sudan or suriname or surinam or dutch guiana or netherlands guiana or syria or syrian arab republic or tajikistan or tadjikistan or tadzhikistan or tadzhik or tanzania or tanganyika or thailand or siam timor leste or east timor or togo or togolese republic or tonga or "trinidad and tobago" or trinidad or tobago or tunisia or turkey or turkmenistan or turkmen or uganda or ukraine or uruguay or uzbekistan or uzbek or vanuatu or new hebrides or venezuela or vietnam or viet nam or middle east or west bank or gaza or palestine or yemen or yugoslavia or zambia or zimbabwe or northern rhodesia or global south or africa south of the sahara or sub-saharan africa or subsaharan africa or africa, central or central africa or africa, northern or north africa or northern africa or magreb or maghrib o sahara or africa, southern or southern africa or africa, eastern or east africa or eastern africa or africa, western or west africa or western africa or west indies or indian ocean islands or caribbean or central america or latin america or "south and central america" or south america or asia, central or central asia or asia, northern or north asia or northern asia or asia, southeastern or southeastern asia or south eastern asia or southeast asia or south east asia or asia, western or western asia or europe, eastern or east europe or eastern europe or developing country or developing countries or developing nation? or developing population? or developing world or less developed countr* or less developed nation? or less developed population or less developed world or lesser developed countr* or lesser developed nation? or lesser developed population? or lesser developed world or under developed countr* or under developed nation? or under developed population? or under developed world or underdeveloped countr* or underdeveloped nation? or underdeveloped population? or underdeveloped world or middle income countr* or middle income nation? or middle income population? or low income countr* or low income nation? or low income population? or lower income countr* or lower income nation? or lower income population? or underserved countr* or underserved nation? or underserved population? or underserved world or under served countr or under served nation? or under served population? or under served world or deprived countr* or deprived nation? or deprived population? or deprived world or poor countr* or poor nation? or poor population? or poor world or poorer countr* or poorer nation? or poorer population? or poor world or developing econom* or less developed econom* or lesser developed econom* or under developed econom* or underdeveloped econom* o middle income econom* or low income econom* or lower income econom* or low gdp or low gnp or low gross domestic or low gross national or low gdp or lower gnp or lower gross domestic or lower gross national or lmic or lmics or third world or lami countr* or transitional countr* or emerging economies or emerging nation?

Concept 2 in Line 2: # hits: 0
youth* or young* or highschool* or "high school*" or student* or adolescent* or peer* or member* or kid* or teen* or "coming of age") NEAR/3 (leader* or design* or facilitat* or develop* or initiat* or coordinat* or serve* or intervention* or initiative* or project* or program* or campaign* or promotion*

Concept 3 in Line 3: # hits: 169, 280

"Maternal Health" OR Pregnancy OR "postpartum period" OR "Peripartum Period" OR pregnan* OR postpartum OR Peripartum OR "Maternal Health" OR "maternal wellbeing" OR "maternal well-being" OR neonat* or newborn or infant or preterm* or prem* or bab*

Line 4: #1 AND #2 AND #3: # hits: 690

CENTRAL (update in 2023)
Database: CENTRAL
Search date: Jan 26, 2023
Applied Search limit/filter: Under Advanced Search>Search Manager
Used same concepts as below, but added filters of:
>>Publication Year from 2022 to 2023
>>Cochrane Library publication date from Jan 2022 to Jan 2023
>>Trials with Pregnancy and Childbirth in Cochrane Groups (Word variations have been searched)
>># hits: 1427 then filtered by:
>custom date: 2022 to 2023
>chose only “Trials”
>># hits screened: 50

Concept 1 in Line 1: # hits: 321133
afghanistan or albania or algeria or american samoa or angola or "antigua and barbuda" or antigua or barbuda or argentina or armenia or armenia aruba or azerbaijan or bahrain or bangladesh or barbados or republic of belarus or belarus or byelarus or belorussia or byelorussian or belize or britis honduras or benin or dahomey or bhutan or bolivia or "bosnia and herzegovina" or bosnia or herzegovina or botswana or bechuanaland or brazil or brasil or bulgaria or burkina faso or burkina fasso or upper volta or burundi or urundi or cabo verde or cape verde or cambodia or kampuchea or khm republic or cameroon or cameron or cameroun or central african republic or ubangi shari or chad or chile or china or colombia or comoros or comoro islands or iles comores or mayotte or democratic republic of the congo or democratic republic congo or congo or zaire or costa rica or "cote divoire" o "cote d ivoire" or cote divoire or cote d ivoire or ivory coast or croatia or cuba or cyprus or czech republic or czechoslovakia or djibouti or french somaliland or dominica or dominican republic or ecuador or egypt or united arab republic or el salvador or equatorial guinea or spanish guinea or eritrea or estonia or eswatini or swaziland or ethiopia or fiji or gabon or gabonese republic or gambia or "georgia (republic) " or georgian or ghana or gold coast or gibraltar or greece or grenada or guam or guatemala or guinea or guinea bissau or guyana or british guiana or haiti or hispaniola or honduras or hungary or india or indonesia or timor or iran or iraq or isle of man or jamaica or jordan or kazakhstan or kazakh or kenya or "democratic peoples republic of korea" or republic of korea or north korea or south korea or korea or kosovo or kyrgyzstan or kirghizia or kirgizstan or kyrgyz republic or kirghiz or laos or lao pdr or "lao people's democratic republic" or latvia or lebanon or lebanese republic or lesotho or basutoland or liberia or libya or libyan arab jamahiriya or lithuania or macau or macao or republic of north macedonia or macedonia or madagascar malagasy republic or malawi or nyasaland or malaysia or malay federation or malaya federation or maldives or indian ocean islands or indian ocean mali or malta or micronesia or federated states of micronesia or kiribati or marshall islands or nauru or northern mariana islands or palau or tuvalu o mauritania or mauritius or mexico or moldova or moldovian or mongolia or montenegro or morocco or ifni or mozambique or portuguese east africa myanmar or burma or namibia or nepal or netherlands antilles or nicaragua or niger or nigeria or oman or muscat or pakistan or panama or papua ne guinea or new guinea or paraguay or peru or philippines or philipines or phillipines or phillippines or poland or "polish people's republic" or portuga or portuguese republic or puerto rico or romania or russia or russian federation or ussr or soviet union or union of soviet socialist republics or rwanda or ruanda or samoa or pacific islands or polynesia or samoan islands or navigator island or navigator islands or "sao tome and principe" or saudi ara or senegal or serbia or seychelles or sierra leone or slovakia or slovak republic or slovenia or melanesia or solomon island or solomon islands or norf island or norfolk islands or somalia or south africa or south sudan or sri lanka or ceylon or "saint kitts and nevis" or "st. kitts and nevis" or saint lucia "st. lucia" or "saint vincent and the grenadines" or saint vincent or "st. vincent" or grenadines or sudan

or suriname or surinam or dutch guiana or netherlands guiana or syria or syrian arab republic or tajikistan or tadjikistan or tadzhikistan or tadzhik or tanzania or tanganyika or thailand or siam timor leste or east timor or togo or togolese republic or tonga or "trinidad and tobago" or trinidad or tobago or tunisia or turkey or turkmenistan or turkmen or uganda or ukraine or uruguay or uzbekistan or uzbek or vanuatu or new hebrides or venezuela or vietnam or viet nam or middle east or west bank or gaza or palestine or yemen or yugoslavia or zambia or zimbabwe or northern rhodesia or global south or africa south of the sahara or sub-saharan africa or subsaharan africa or africa, central or central africa or africa, northern or north africa or northern africa or magreb or maghrib o sahara or africa, southern or southern africa or africa, eastern or east africa or eastern africa or africa, western or west africa or western africa or west indies or indian ocean islands or caribbean or central america or latin america or "south and central america" or south america or asia, central or central asia or asia, northern or north asia or northern asia or asia, southeastern or southeastern asia or south eastern asia or southeast asia or south east asia or asia, western or western asia or europe, eastern or east europe or eastern europe or developing country or developing countries or developing nation? or developing population? or developing world or less developed countr* or less developed nation? or less developed population or less developed world or lesser developed countr* or lesser developed nation? or lesser developed population? or lesser developed world or under developed countr* or under developed nation? or under developed population? or under developed world or underdeveloped countr* or underdeveloped nation? or underdeveloped population? or underdeveloped world or middle income countr* or middle income nation? or middle income population? or low income countr* or low income nation? or low income population? or lower income countr* or lower income nation? or lower income population? or underserved countr* or underserved nation? or underserved population? or underserved world or under served countr or under served nation? or under served population? or under served world or deprived countr* or deprived nation? or deprived population? or deprived world or poor countr* or poor nation? or poor population? or poor world or poorer countr* or poorer nation? or poorer population? or poor world or developing econom* or less developed econom* or lesser developed econom* or under developed econom* or underdeveloped econom* o middle income econom* or low income econom* or lower income econom* or low gdp or low gnp or low gross domestic or low gross national or low gdp or lower gnp or lower gross domestic or lower gross national or lmic or lmics or third world or lami countr* or transitional countr* or emerging economies or emerging nation?

Concept 2 in Line 2: # hits: 0
youth* or young* or highschool* or "high school*" or student* or adolescent* or peer* or member* or kid* or teen* or "coming of age") NEAR/3 (leader* or design* or facilitat* or develop* or initiat* or coordinat* or serve* or intervention* or initiative* or project* or program* or campaign* or promotion*

Concept 3 in Line 3: # hits: 188416
"Maternal Health" OR Pregnancy OR "postpartum period" OR "Peripartum Period" OR pregnan* OR postpartum OR Peripartum OR "Maternal Health" OR "maternal wellbeing" OR "maternal well-being" OR neonat* or newborn or infant or preterm* or prem* or bab*

Line 4: #1 AND #2 AND #3: # hits: 1427>>>50 hits with filters of date range and as only "trials"

WEBSITES
US National Institutes of Health Ongoing Trials Register Clinical Trials.gov
Database: ClinicalTrials.gov
Search date: Feb 2022 and Jan 28, 2023
Search terms>>Completed Studies | Interventional Studies | maternal death (as condition or disease)
Applied Filters: Study results: Completed studies, Study type: Interventional studies
# of hits: 29 studies
Scanned titles from list; if title seemed appropriate, trial complete, results available and done in LMIC then screened by abstract>>2 studies screened by abstract and other study details
None kept

World Health Organization (WHO) International Clinical Trials Registry Platform (WHO ICTRP)
Database: WHO ICTRP
Search date: Jan 25, 2022
Initial search:
Concept 1 (title): low and middle-income countries
Concept 2 (condition): maternal death OR neonatal death
Concept 3 (intervention): youth* or young* or highschool* or "high school*" or student* or adolescent* or peer* or member* or kid* or teen* or "coming of age") NEAR/3 (leader* or design* or facilitat* or develop* or initiat* or coordinat* or serve* or intervention* SINCE limited by # of characters
# of hits: 0

Adapted search:
1) List By>List By Health Topic>Maternal health--------86 trials
2) List By>List By Health Topic> Neonatal health-------34 trials
3) List By>List By Health Topic>Reproductive health----1501 trials
4) List By>List By Health Topic>Women's health----118 trials

Total # screened manually; none kept

WHO ICTRP (update in 2023)
Database: WHO ICTRP
Search date: Jan 26, 2023
Initial search:
Concept 1 (title): low and middle-income countries
Concept 2 (condition): maternal death OR neonatal death
Concept 3 (intervention): youth* or young* or highschool* or "high school*" or student* or adolescent* or peer* or member* or kid* or teen* or "coming of age") NEAR/3 (leader* or design* or facilitat* or develop* or initiat* or coordinat* or serve* or intervention* SINCE limited by # of characters
# of hits: 0

Adapted search:
1) List By>List By Health Topic>Maternal health--------3 trials with results (of 96 hits) "manually" reviewed at T/A level/checking trial websites>>>0/3 kept
2) List By>List By Health Topic> Neonatal health-------1 trial with results "manually" reviewed at T/A level/checking trial websites>>>0/1 kept
3) List By>List By Health Topic>Reproductive health------86 trials with results (of 1582 hits) "manually" reviewed at T/A level/checking trial websites>>>0/86
4) List By>List By Health Topic>Women's health------did not run this search as expected most studies would be captured by other searches

ISRCTN Registry (Biomed Central)
Database:
https://www.isrctn.com/search?q=maternal+health+interventions+in+low+and+middle+income+countries&searchType=advanced-search
Search date: Apr 8, 2022
Search terms: maternal health interventions in low and middle income countries
# of hits: 13; none kept

ISRCTN Registry (update in 2023)
Database:
https://www.isrctn.com/search?q=maternal+health+interventions+in+low+and+middle+income+countries&searchType=advanced-search
Search date: Jan 26, 2023

Search terms: maternal health interventions in low and middle income countries
>>Filter: Trial completed
# of hits: 10; none kept

ORGANIZATIONS
World Health Organization Health Evidence Network (WHO HEN)
Database: https://www.who.int/europe/publications/i
Search date: February 2022 only
Search Terms: maternal health interventions in low and middle income countries
Used filters:
>>maternal, newborn, child and adolescent health
>>all publication types, all countries, all years, all publishing offices
# of hits: 0; none kept

Centers for Disease Control and Prevention (CDC)
Database: https://search.cdc.gov/search/?query=database&dpage=1
Search date: February 2022 only
Search Terms: advanced search using: maternal health interventions in low and middle income countries + journal filter
# of hits: 0; none kept

GREY LITERATURE
Directory of Open Access Journals (DOAJ)
Database: https://doaj.org/
Search date: April 7, 2022
Search terms: maternal health interventions in low and middle income countries
# of hits: 244
*3 studies manually screened as full text; none kept

DOAJ (update in 2023)
Database: https://doaj.org/
Search date: Jan 26, 2023
Search terms: maternal health interventions in low and middle income countries
# of hits: 296
>>Added filter: Year of publication
2022: #of hits: 57 >>>TM manually screened T/A online; NONE retained
2023: #of hits: 3 >>> TM manually screened T/A online; NONE retained
Therefore total # of hits screened: 60; none kept

National Institute of Health Research (NIHR)
Database: https://www.io.nihr.ac.uk/outputs-publications/
Search date: April 8, 2022
Search terms: used filters Sexual Health, Pregnancy and Childbirth
# of hits: 116; manually screened; none kept

NIHR (update in 2023)
Database: https://www.io.nihr.ac.uk/outputs-publications/
Search date: Jan 26, 2023
Search terms: maternal health interventions in low and middle income countries
>>Post type: Journal article
>>Technology category: Methods

>>Year: 2022 and then 2023
# of hits for 2022 and then 2023: 0; none kept

Appendix S3
Data Items

- The report: authors, year, and source of publication, ethics approval, funding statement
- The study trial characteristics: study design, single or multi-centre, country, country's development criteria per WHO, country's income level per World Bank economic categorization, language of publication
- The participants characteristics (at baseline): age, gender, education level (or literacy), asset score quintile, race or ethnicity, antenatal care, parity, duration of residence, other (e.g., religion), eligibility and exclusion criteria used in the study
- The characteristics of the youth-led intervention: name of intervention, number of participants randomised and analysed; provider's age for delivery of intervention, components of intervention, how youth-led, if adherence evaluated; number, duration, frequency of sessions, time in months of entire intervention and time points when measurements were taken
- The characteristics of the comparator group: name of control, provider's profession, if training was provided; number of participants randomised and analysed; number, duration, frequency of sessions, time in months of entire comparator and time points when measurements were taken

Appendix S4
Details of Four Included Studies
Details of the four included study are described below and include information about study design, setting, sampling, participants, study aim, intervention, youth involvement in intervention, control, and outcomes.

Study 1 [7]**:** The study by Gullo et al. is a **cluster RCT** conducted in Ntcheu district of rural Malawi. The **sample size** consisted of 20 clusters (2601 women) in which two-stage cluster sampling was used to select group villages and villages for participation in the study.

P**articipants** consisted of 2601 enrolled individuals. These were women from village clusters, aged 15-49, who had given birth within the last 12 months (since Nov 2012) and whose baby was still living. The **study aim** was to evaluate the effects of a social accountability approach on reproductive health outcomes.

The **intervention**, the CARE Community Score Card (CSC), is a social mobilisation and behaviour change intervention. This social accountability tool aimed to empower community members, healthcare providers and other government stakeholders to identify and overcome obstacles to health coverage, quality, and equity in resource-limited settings. There was a 5-phase CSC process to the intervention that involved: 1) planning/preparation 2) conducting CSC with the community 3) conducting CSC with service providers 4) interface meeting and action planning 5) action plan implementation and monitoring. The intervention was delivered by Facility-based service providers, Community Health Workers, Health Surveillance Assistants, and community members.

Youth community members (aged 15-30) were involved at different points of CSC intervention. The intervention was delivered every six to seven months, to include six sessions (of various durations) over a 24-month period. Youth were consulted for their input before the study was launched. Subsequently their involvement included administration of the intervention and knowledge translation/mobilisation activities. Ten clusters received the CSC intervention which included 1303 individuals who were randomised, and 651 who were analyzed.

The **control** groups consisted of ten clusters receiving standard reproductive health services delivered by Facility-based service providers and included 1298 randomised to the control, and 649 who were analyzed.

The trial's primary **outcomes** included: modern contraceptive use, antenatal and postnatal care service utilization, and service satisfaction. There were no review primary outcomes reported. But our secondary outcome of ANC coverage (at least four antenatal visits) was reported in the trial. We used the trial's sufficient antenatal care received (4+ visits) and examined these in the **follow-up** measured at 24 months.

Study 2 [8]**:** The study by Cowan et al. is a **cluster RCT** conducted in the communities of seven rural districts in south-eastern Zimbabwe. The **sample size** comprised 30 clusters where communities were randomised using restricted randomisation. In the final survey, enumeration areas were purposively selected to ensure that sites where intervention activities took place in that community were included.

There were 6791 enrolled individuals, of which 2593 were women (56% of enrolled participants). Eligible **participants** were young people in or out of secondary school, and all 18–22-year-olds living in the 180 enumeration areas of the identified cluster communities. The **study aim** was to determine the effectiveness of a community-based multicomponent HIV and reproductive health intervention.

The Regai Dzive Shiri (RDS) **intervention** is a three-component bundled intervention of social mobilisation, education/communication, and behaviour change. The intervention is aimed at changing societal norms within communities and is delivered to young people, parents, and clinic staff.

Youth involved in the intervention consisted of those who were "school leavers" (i.e., completed secondary school). These youth as professional peer educators (PPEs) played a role in administration of all three

components of intervention. As PPEs they led the in-school program by using participatory methods to deliver materials aimed at enhancing knowledge and developing skills. They were also involved in the out-of-school program that consisted of 24 education-behaviour change sessions over four weeks. Fifteen clusters received RSD intervention which included 3381 randomised women and men, and 2319 individuals who were analyzed.

The **control** groups comprised 15 clusters who received a "deferred intervention" consisting of standard HIV prevention activities (in-school guidance, counselling sessions, testing) delivered once per month by staff of District AIDS Action Committees. There were 3410 women and men randomised to the control, and 2353 who were analyzed.

The trial's primary **outcomes** were biological outcomes of HIV and HSV-2 infections. Secondary outcomes were pregnancy prevalence, and reported knowledge, behaviour and attitudes. The only reported outcome that corresponded to our systematic review was secondary outcome (youth) pregnancy which we considered a proxy to Adolescent Birth. We used Currently pregnant (see Cowan et al., Table 5). We calculated this as "youth" (age 15-24) vs adolescent pregnancy since we could not ascertain disaggregated ages of those currently pregnant. **Follow-up** of the outcome measures occurred at 36 and 48 months.

Study 3 [9]**:**The study by Ross et al. is a **cluster RCT** conducted in 20 well-separated rural communities in Mwanza region of Tanzania. The **sample size** consisted of 20 clusters. Clusters were formed into three risk strata based on data from a previous population-based survey, and arms were balanced for HIV and chlamydia prevalence using restricted randomisation. Ten communities received the intervention, and 10 communities were the comparison groups. There were 9219 individuals recruited, enrolled and randomised into the study in late 1998, although and an additional 426 individuals were recruited and randomised at the interim follow-up in 2000 (i.e., total 9645 enrolled in study). The 4116 women participants made up 44.6% of those randomised.

Eligible **participants** were all adolescents (male and female) aged 14 years of more in late 1998, who were in years 4-6 (and about to enter years 5-7; i.e., 14-18+ years old) of the 121 government primary schools within the 20 cluster communities. The **study aim** was to evaluate the impact of the MEMA kwa Vijana ('Good things for young people') intervention on HIV incidence, prevalence/incidence of other STI and pregnancy, and sexual health knowledge, attitudes and reported sexual behaviour.

The MEMA kwa Vijana **intervention** consisted of four major components: 1) a participatory, teacher-led and peer-assisted in-school program 2) provision of youth-friendly sexual and reproductive health services by health workers 3) community-based condom promotion and distribution by youth and 4) community-wide mobilisation activities and youth-focussed events (e.g., health week, video shows) led by community members including youth. The in-school program component had twelve 40-minute sessions within the school year.

Youth involvement occurred in three components of the intervention: in-school program as peer-assistants in sexual health education, during peer condom social marketing and distribution, and a part of community-wide activities. As peer-educators, for example, youth peer-educators performed brief dramas as discussion starters among their schoolmates.

The **control** groups consisted of ten clusters that received standard sexual health activities which were reported to be very limited and similar to non-MEMA kwa Vijana activities. There were 4775 males and females randomised to the control, and 3516 were analyzed. Standard activities (carried out by teachers and health workers) included family planning services, improved case management of sexually transmitted infections (STIs).

The trial's primary **outcomes** were HIV seroincidence during follow-up and HSV2 seroprevalence at final survey. Secondary outcomes included additional biological, behavioural, attitudinal and knowledge

outcomes related to HIV, STI acquisition, and attitudes towards sex and sexual behaviours, as well as biological outcomes like STI prevalence and pregnancy. For review outcomes reported, there were no primary ones. We considered pregnancy (among adolescents, i.e., those aged 15-19 years old) as a secondary outcome and a proxy to Adolescent Birth. We used "Reported pregnancy during follow-up" from Table 3 (Ross et al.) which includes those from Years 4-6 who were 17 years old+ to 19 years old at the final survey. **Follow-up** outcomes were measured at 36 months.

Study 4 [10]**:** The study by Jewkes et al. is a **parallel cluster RCT** conducted in 70 communities (64 villages and six townships) in rural South Africa. The **sample size** comprised 70 clusters (1416 women, i.e., 51.0% of 2776 enrolled and randomised) of 35 clusters per trial arm. Clusters were grouped into seven strata; and within each stratum equal numbers of clusters were allocated to intervention and control arms. Determination of the number of clusters was based on an 80% power calculation to detect a 5% significance level for a 50% reduction in HIV incidence.

Eligible **participants** were individuals aged 15-26 years who were mostly attending school. Of the 2776 enrolled individuals in the study, there were 1360 men and 1416 women. The **study aim** was to assess the impact of the South African Stepping Stones HIV prevention program (second edition) on the incidence of HIV and HSV-2 and sexual practices among men and women in rural areas in the Eastern Cape province of South Africa.

The South African Stepping Stones **intervention** was received by 35 clusters (females and males: 1409 randomised, 1140 analyzed). This intervention was adapted for the South African context and contained participatory sessions of single sex groups, run in parallel. This comprised thirteen three-hour sessions, three peer group meetings, and one community meeting. The duration of sessions was a total of 50 hours over a six-to-eight-week period. These allowed for critical reflection, roleplay, and drama and drew on the everyday reality of participants' lives. According to attendance registers, 59.1% women attended at least 75% or greater of the intervention sessions; and 25.4% of women attended the complete program.

Youth involvement consisted of non-governmental organization project staff who were young people aged 16-23+ years. These youth were trained as intervention Facilitators that included three weeks of training and two practice groups. Youth Facilitators were matched to study participants who were of the same sex and (approximate) age.

Thirty-five clusters received a "**control** intervention" that consisted of one-time three-hour intervention on HIV, safer sex, and condoms (females and males: 1367 randomised to control, 1081 analyzed). Other youth Facilitators carried out the control activities after receiving four days of training; they were same sex and (approximate) age as study participants.

The trial's primary **outcome** was incidence of HIV. Secondary outcomes included incidence of HSV-2, unwanted pregnancy, reported sexual practices, depression, and substance misuse. . For review outcomes reported, there were no primary ones. We considered secondary outcome "unwanted pregnancy". We calculated this as youth "unwanted pregnancy" vs adolescent "unwanted pregnancy" since we could not ascertain disaggregated ages of participants currently with an "unwanted pregnancy" measured at 24 months. We took youth unwanted pregnancy as a proxy for youth birth. **Follow-up** outcomes were taken at 12 and 24 months.

Table S1: TIDieR Intervention Summary

AUTHOR (year)	BRIEF NAME	WHY	WHAT (materials)	WHAT (procedures)	WHO PROVIDED	HOW	WHERE	WHEN and HOW MUCH	TAILORING	MODIFICATIONS	HOW WELL (planned strategies)	HOW WELL (actual delivery)
**Gullo** (2017)	CSC: complex social account-ability tool	To empower community members, HCPs and govt officials to identify and overcome obstacles to health coverage, quality and equity in resource-limited settings	Community-developed CSC; identified issues, themes, and measurable indicators/ themes via 5-phases: 1) plan 2) conduct CSC w community 3) conduct CSC w FBSPs 4) interface mtg and action planning 5) action plan implementa-tion and monitoring	CSC developed w 13 indicators; actions to improve scores agreed upon through FGDs; 5-phase process repeated	FBSPs, CHWs, HSAs, community members (men, women and youth); youth involved at different points	FGDs w community, in parallel w FBSPs; interface mtg w govt officials, action implementation, M+E of indicators q3 mo; delivered in community face-to-face; as 40-60 min survey	Health facilities and community	Repeated 4 of 5-phase CSC development cycle q6-7 mo over 24 mo; 6 sessions; varied duration	When SS could not be met, interviewers went to nearest village to complete data collection	Not described	Not described	NA
**Cowan** (2010)	RDS: bundled intervention of social mobilisation, education, communica-tion, behaviour change w biological endpoints as primary outcomes; Social Learning Theory, and Stages of	To change societal norms within communities about issues of HIV and reproductive health	Theoretically based. Used participatory methods to enhance knowledge and develop skills	Intervention delivered via 3 programs to: 1. Youth in trial schools and community 2. Parents and community stakeholders 3. Nurses and other staff in youth-friendly rural clinics	1. PPEs delivered to in/out-of-school youth 2. Project facilitators to parents, community stakeholders via community-based sessions 3. Project facilitators to nurses and other clinic staff	All programs delivered face-to-face, in group settings. PPEs involved in delivery of components of all 3 programs; audio-SAQ and ACASI, and biochemical tests (blood sample and urine test for pregnancy)	1. Trial schools; out-of-school program in community 2. In community; not specified 3. Presumably at clinics; to improve clinic accessibility for young people	1. In trial schools, throughout school year; 24-sessions in 4 wk for out-of-school program 2. In community via 22-session program 3. Not described except: to improve clinic accessibility for young people	Intervention became increasingly community-based (due to drop in school attendance). Broadened original school-going cohort survey to population-based survey. For final survey, enumeration	Conducted cross-sectional population-based survey given decline in HIV incidence in country, need to revise study SS, and due to population mobility (outmigration) during trial	Intervention adherence via registry of attendance at sessions, with minimum of 75% attendance of sessions*	Adherence: 1. 30% in trial schools; 20% in out-of-school program attended 10+ sessions; 41% attended either in/out-of school program 2. In community: not specified

AUTHOR (year)	BRIEF NAME	WHY	WHAT (materials)	WHAT (procedures)	WHO PROVIDED	HOW	WHERE	WHEN and HOW MUCH	TAILORING	MODIFICATIONS	HOW WELL (planned strategies)	HOW WELL (actual delivery)
	Change Model				All carefully selected, trained and supervised				areas purposively selected to ensure that sites where intervention activities took place in that community were included			3. At clinics: not specified
**Ross** (2010)	*MEMA kwa Vijana* "Good things for young people': Multi-component program on adolescent sexual health	To improve adolescent sexual health knowledge, attitudes and sexual behaviour; see improvement in HIV and other STI incidence, and pregnancy	Built on experience of other reproductive health projects, i.e., with 10 character-istics of effective programs	4 major components:<br>1.Participatory, teacher-led and peer-assisted program<br>2. Youth-friendly sexual and reproductive health services<br>3. Condom social marketing and distribution<br>4. Community activities and youth-focused events	1. Teachers and Youth/Peer Assistants<br>2. Health workers stakeholders<br>3. Youth<br>4. Community members including youth	All programs delivered face-to-face, in groups or individually.<br>1. Brief dramas, discussions<br>2. SRHS including FP services and case management of STI<br>3. Condom distribution<br>4. Featured youth health weeks/days, videos, all linked to discussions	1. Primary schools<br>2. Health clinics<br>3.Commun-ity<br>4. Commun-ity	1. One 40-min session/mo over 12 mo of school year<br>2. One wks' training in provision of youth-friendly SRHS<br>3. Youth elected by peers and trained in social marketing of condoms<br>4. Initial community mobilization, then annual health weeks, 2x/yr health days, 4x/yr video shows	Interviews done by same-sex, 20-24 yo RAs. Clinical checks by same-sex as participant to assess for S+S of STI; and offered HIV counselling and testing<br><br>Efforts to locate cohort members included up to 6 household visits and attempts to trace out-migrants	Not described	1. Teacher training, and youth supervised by teacher; quarterly supervision visits<br>2. Trained and supervised quarterly<br>3. Trained youth; initiated in response to results of process evaluation<br>4. Commun-ity	Extensive process evaluation of intervention described as delivered to high standard and with high coverage.<br>1. Over 80% of sessions had been taught 2-3 mo before end of each school yr during trial<br>2. Increased respect and empathy to youth<br>3. Over 57500 condoms sold in 2 yrs of condom distribution
**Jewkes** (2008)	SSSA: participatory HIV prevention program on sexual	To improve sexual health by participatory learning approaches,	SSSA program activity materials	Adapted to South Africa: participatory sessions, single sex	Facilitators were project staff, employed by partner NGO; trained, supervised and shown how to	Delivered face-to-face, in single sex grs, peer gr mtgs, community mtg. Included critical reflection, roleplay and drama.	Mainly held on school premises after school hours	Program spanned about 50 h (ran for 6-8 wks)<br>Included: 13 three-h sessions, 3 peer gr mtgs,1 community mtg	Participants could change their mind about being told their HIV	Change to consent form when anti-retroviral drugs became publicly available; changed to ask those opting	Attendance registers for attendance of complete program (although an	Adherence: 16.8% men and 12.5% women did not participate in

AUTHOR (year)	BRIEF NAME	WHY	WHAT (materials)	WHAT (procedures)	WHO PROVIDED	HOW	WHERE	WHEN and HOW MUCH	TAILORING	MODIFICATIONS	HOW WELL (planned strategies)	HOW WELL (actual delivery)
		and through building stonger, more gender equitable relationships; aims to build knowledge, risk awareness, and communica-tion skills, and to stimulate critical reflection		groups run in parallel	implement program; same sex as participants, and approximately same age. Selected in part for their open mindedness and gender sensitivity				testing results and get their results at any stage; participants w social problems and HIV related problems	not to collect their HIV result if they would like to know their status if testing was positive	incomplete set of registers)	any sessions. 60.7% men and 59.1% women attended 75%+ of sessions. 27.5% men and 25.4% women attended complete program

ACASI, audio-computer-assisted survey instrument; audio-SAQ, audio-self-administered questionnaire; CHW, community health worker; CSC, community score card; FBSP, facility-based service provider; FGD, focus group discussion; FP, family planning; govt, government; gr, group; h, hours; HCP, healthcare providers; HSA, Health Surveillance Assistant; HIV, human immunodeficiency virus; HSV2, herpes simplex virus-2; M+E, monitoring and evaluation; min, minute; mo, months; mtg, meeting; NA, not applicable; NGO, non-governmental organization; NR, not recorded; pp, postpartum; PPE, professional peer educator; q, every; RA, research assistant; RDS, Regai Dzive Shiri; SRHS, sexual and reproductive health services; SS, sample size; S+S, signs and symptoms; SSSA, Stepping Stones South Africa; STI, sexually transmitted infection; w, with; wk, week; yo, year old

*Data from [11]; Table adapted from [12]

Table S2: PRISMA 2020 for Abstracts Checklist

Section and Topic	Item #	Checklist item	Reported (Yes/No)
**TITLE**			
Title	1	Identify the report as a systematic review.	Yes
**BACKGROUND**			
Objectives	2	Provide an explicit statement of the main objective(s) or question(s) the review addresses.	Yes
**METHODS**			
Eligibility criteria	3	Specify the inclusion and exclusion criteria for the review.	Yes
Information sources	4	Specify the information sources (e.g., databases, registers) used to identify studies and the date when each was last searched.	Yes
Risk of bias	5	Specify the methods used to assess risk of bias in the included studies.	Yes
Synthesis of results	6	Specify the methods used to present and synthesise results.	Yes
**RESULTS**			
Included studies	7	Give the total number of included studies and participants and summarise relevant characteristics of studies.	Yes
Synthesis of results	8	Present results for main outcomes, preferably indicating the number of included studies and participants for each. If meta-analysis was done, report the summary estimate and confidence/credible interval. If comparing groups, indicate the direction of the effect (i.e., which group is favoured).	Yes
**DISCUSSION**			
Limitations of evidence	9	Provide a brief summary of the limitations of the evidence included in the review (e.g., study risk of bias, inconsistency and imprecision).	Yes
Interpretation	10	Provide a general interpretation of the results and important implications.	Yes
**OTHER**			
Funding	11	Specify the primary source of funding for the review.	Yes
Registration	12	Provide the register name and registration number.	Yes

Source of table: [13]

For more information, visit: http://www.prisma-statement.org

Table S3: PRISMA 2020 Checklist

Section and Topic	Item #	Checklist item	Location where item is reported
**TITLE**			
Title	1	Identify the report as a systematic review.	Pgs 1 and 2
**ABSTRACT**			
Abstract	2	See the PRISMA 2020 for Abstracts checklist.	Pg 2
**INTRODUCTION**			
Rationale	3	Describe the rationale for the review in the context of existing knowledge.	Pg 3
Objectives	4	Provide an explicit statement of the objective(s) or question(s) the review addresses.	Pg 4
**METHODS**			
Eligibility criteria	5	Specify the inclusion and exclusion criteria for the review and how studies were grouped for the syntheses.	Pg 4
Information sources	6	Specify all databases, registers, websites, organisations, reference lists and other sources searched or consulted to identify studies. Specify the date when each source was last searched or consulted.	Pgs 4-5
Search strategy	7	Present the full search strategies for all databases, registers and websites, including any filters and limits used.	Pg 5
Selection process	8	Specify the methods used to decide whether a study met the inclusion criteria of the review, including how many reviewers screened each record and each report retrieved, whether they worked independently, and if applicable, details of automation tools used in the process.	Pg 5
Data collection process	9	Specify the methods used to collect data from reports, including how many reviewers collected data from each report, whether they worked independently, any processes for obtaining or confirming data from study investigators, and if applicable, details of automation tools used in the process.	Pg 5
Data items	10a	List and define all outcomes for which data were sought. Specify whether all results that were compatible with each outcome domain in each study were sought (e.g., for all measures, time points, analyses), and if not, the methods used to decide which results to collect.	Pgs 5-6
	10b	List and define all other variables for which data were sought (e.g., participant and intervention characteristics, funding sources). Describe any assumptions made about any missing or unclear information.	Pgs 5-6
Study risk of bias assessment	11	Specify the methods used to assess risk of bias in the included studies, including details of the tool(s) used, how many reviewers assessed each study and whether they worked independently, and if applicable, details of automation tools used in the process.	Pg 6
Effect measures	12	Specify for each outcome the effect measure(s) (e.g., risk ratio, mean difference) used in the synthesis or presentation of results.	Pg 6
Synthesis methods	13a	Describe the processes used to decide which studies were eligible for each synthesis (e.g., tabulating the study intervention characteristics and comparing against the planned groups for each synthesis (item #5)).	Pg 6
	13b	Describe any methods required to prepare the data for presentation or synthesis, such as handling of missing summary statistics, or data conversions.	Pg 6
	13c	Describe any methods used to tabulate or visually display results of individual studies and syntheses.	Pg 6
	13d	Describe any methods used to synthesize results and provide a rationale for the choice(s). If meta-analysis was performed, describe the model(s), method(s) to identify the presence and extent of statistical heterogeneity, and software package(s) used.	Pg 6
	13e	Describe any methods used to explore possible causes of heterogeneity among study results (e.g., subgroup analysis, meta-regression).	Pg 7
	13f	Describe any sensitivity analyses conducted to assess robustness of the synthesized results.	Pg 7

Section and Topic	Item #	Checklist item	Location where item is reported
Reporting bias assessment	14	Describe any methods used to assess risk of bias due to missing results in a synthesis (arising from reporting biases).	Pg 7
Certainty assessment	15	Describe any methods used to assess certainty (or confidence) in the body of evidence for an outcome.	Pg 7
**RESULTS**			
Study selection	16a	Describe the results of the search and selection process, from the number of records identified in the search to the number of studies included in the review, ideally using a flow diagram.	Pg 7, Fig. 1
	16b	Cite studies that might appear to meet the inclusion criteria, but which were excluded, and explain why they were excluded.	Pg 7
Study characteristics	17	Cite each included study and present its characteristics.	Pg 7, Table 1, Appendices S4 and Table S1
Risk of bias in studies	18	Present assessments of risk of bias for each included study.	Pgs 7-8, Table 2
Results of individual studies	19	For all outcomes, present, for each study: (a) summary statistics for each group (where appropriate) and (b) an effect estimate and its precision (e.g. confidence/credible interval), ideally using structured tables or plots.	Pgs 8-9
Results of syntheses	20a	For each synthesis, briefly summarise the characteristics and risk of bias among contributing studies.	Pg 9, Fig. 2
	20b	Present results of all statistical syntheses conducted. If meta-analysis was done, present for each the summary estimate and its precision (e.g., confidence/credible interval) and measures of statistical heterogeneity. If comparing groups, describe the direction of the effect.	Pg 9
	20c	Present results of all investigations of possible causes of heterogeneity among study results.	Pg 9
	20d	Present results of all sensitivity analyses conducted to assess the robustness of the synthesized results.	Pg 9
Reporting biases	21	Present assessments of risk of bias due to missing results (arising from reporting biases) for each synthesis assessed.	Pg 9
Certainty of evidence	22	Present assessments of certainty (or confidence) in the body of evidence for each outcome assessed.	Pg 9, Table 3
**DISCUSSION**			
Discussion	23a	Provide a general interpretation of the results in the context of other evidence.	Pgs 9-10
	23b	Discuss any limitations of the evidence included in the review.	Pg 10
	23c	Discuss any limitations of the review processes used.	Pg 10
	23d	Discuss implications of the results for practice, policy, and future research.	Pg 10
**OTHER INFORMATION**			
Registration and protocol	24a	Provide registration information for the review, including register name and registration number, or state that the review was not registered.	Pg 11
	24b	Indicate where the review protocol can be accessed, or state that a protocol was not prepared.	Pg 11
	24c	Describe and explain any amendments to information provided at registration or in the protocol.	Pg 11
Support	25	Describe sources of financial or non-financial support for the review, and the role of the funders or sponsors in the review.	Pg 11
Competing interests	26	Declare any competing interests of review authors.	Pg 11

Section and Topic	Item #	Checklist item	Location where item is reported
Availability of data, code and other materials	27	Report which of the following are publicly available and where they can be found: template data collection forms; data extracted from included studies; data used for all analyses; analytic code; any other materials used in the review.	Pg 11 and Appendix S2

Source of table: [13]

For more information, visit: http://www.prisma-statement.org/

References

1 World Health Organization (WHO). Trends in maternal mortality 2000 to 2017: estimates by WHO, UNICEF, UNFPA, World Bank Group and the United Nations Population Division. Geneva: World Health Organization, 2019.

2 World Health Organization (WHO). The global health observatory: indicator metadata registry list: neonatal mortality rate (per 1000 live births). 2021. Available: https://www.who.int/data/gho/indicator-metadata-registry/imr-details/67. Accessed: 24 September 2021.

3 World Health Organization (WHO). The global health observatory: indicator metadata registry list: antenatal care coverage- at least four visits. 2021. Available: https://www.who.int/data/gho/indicator-metadata-registry/imr-details/80. Accessed: 24 September 2021.

4 World Health Organization (WHO). Health topics: stillbirth. 2021. Available: https://www.who.int/health-topics/stillbirth#tab=tab_1. Accessed: 20 October 2021.

5 World Health Organization (WHO). Programmes: sexual reproductive health: WHO recommendations on prevention and treatment of postpartum haemorrhage and the WOMAN trial. 2021. Available: https://www.who.int/reproductivehealth/topics/maternal_perinatal/pph-woman-trial/en/. Accessed: 20 October 2021.

6 World Health Organization (WHO). Evaluating the quality of care for severe pregnancy complications: the WHO near-miss approach for maternal health. Geneva: World Health Organization, 2011.

7 Gullo S, Galavotti C, Kuhlmann AS, Msiska T, Hastings P, Marti CN. Effects of a social accountability approach, CARE's Community Score Card, on reproductive health-related outcomes in Malawi: A cluster-randomized controlled evaluation. PLoS One. 2017;12.

8 Cowan FM, Pascoe SJS, Langhaug LF, Mavhu W, Chidiya S, Jaffar S, et al. The Regai Dzive Shiri project: results of a randomized trial of an HIV prevention intervention for youth. AIDS. 2010;24:2533-44.

9 Ross DA, Changalucha J, Obasi AI, Todd J, Plummer ML, Cleophas-Mazige B, et al. Biological and behavioural impact of an adolescent sexual health intervention in Tanzania: a community-randomized trial. AIDS. 2007;21:1943-55.

10 Jewkes R, Nduna M, Levin J, Jama N, Dunkle K, Puren A, et al. Impact of Stepping Stones on incidence of HIV and HSV-2 and sexual behaviour in rural South Africa: cluster randomised controlled trial. BMJ. 2008;337:a506.

11 Cowan FM, Pascoe SJS, Langhaug LF, Dirawo J, Chidiya S, Jaffar S, et al. The Regai Dzive Shiri Project: a cluster randomised controlled trial to determine the effectiveness of a multi-component community-based HIV prevention intervention for rural youth in Zimbabwe - study design and baseline results. Trop Med Int Health. 2008;13:1235-44.

12 Hoffmann TC, Glasziou PP, Boutron I, Milne R, Perera R, Moher D, et al. Better reporting of interventions: template for intervention description and replication (TIDieR) checklist and guide. BMJ. 2014;348:g1687.

13 Page MJ, McKenzie JE, Bossuyt PM, Boutron I, Hoffmann TC, Mulrow CD, et al. The PRISMA 2020 statement: an updated guideline for reporting systematic reviews. BMJ. 2021;372:n71.
